# Inhibited complete folding of consecutive human telomeric G-quadruplexes

**DOI:** 10.1093/nar/gkad004

**Published:** 2023-01-30

**Authors:** Emil Laust Kristoffersen, Andrea Coletta, Line Mørkholt Lund, Birgit Schiøtt, Victoria Birkedal

**Affiliations:** Interdisciplinary Nanoscience Center (iNANO), Aarhus University, Gustav Wieds Vej 14, 8000 Aarhus, Denmark; Department of Chemistry, Aarhus University, Langelandsgade 140, 8000 Aarhus, Denmark; Interdisciplinary Nanoscience Center (iNANO), Aarhus University, Gustav Wieds Vej 14, 8000 Aarhus, Denmark; Interdisciplinary Nanoscience Center (iNANO), Aarhus University, Gustav Wieds Vej 14, 8000 Aarhus, Denmark; Department of Chemistry, Aarhus University, Langelandsgade 140, 8000 Aarhus, Denmark; Interdisciplinary Nanoscience Center (iNANO), Aarhus University, Gustav Wieds Vej 14, 8000 Aarhus, Denmark; Department of Chemistry, Aarhus University, Langelandsgade 140, 8000 Aarhus, Denmark

## Abstract

Noncanonical DNA structures, termed G-quadruplexes, are present in human genomic DNA and are important elements in many DNA metabolic processes. Multiple sites in the human genome have G-rich DNA stretches able to support formation of several consecutive G-quadruplexes. One of those sites is the telomeric overhang region that has multiple repeats of TTAGGG and is tightly associated with both cancer and aging. We investigated the folding of consecutive G-quadruplexes in both potassium- and sodium-containing solutions using single-molecule FRET spectroscopy, circular dichroism, thermal melting and molecular dynamics simulations. Our observations show coexistence of partially and fully folded DNA, the latter consisting of consecutive G-quadruplexes. Following the folding process over hours in sodium-containing buffers revealed fast G-quadruplex folding but slow establishment of thermodynamic equilibrium. We find that full consecutive G-quadruplex formation is inhibited by the many DNA structures randomly nucleating on the DNA, some of which are off-path conformations that need to unfold to allow full folding. Our study allows describing consecutive G-quadruplex formation in both nonequilibrium and equilibrium conditions by a unified picture, where, due to the many possible DNA conformations, full folding with consecutive G-quadruplexes as beads on a string is not necessarily achieved.

## INTRODUCTION

DNA metabolic processes such as replication, transcription, telomere maintenance and DNA repair are essential for cellular survival. Hundreds of thousands of potential and confirmed G-quadruplex-forming motives have been identified throughout the human genomic DNA and RNA (1–6) and are highly conserved between species (7–9). These motives consist of guanine-rich DNA able to form stable long-lived G-quadruplex structures and are found in DNA replication origins, gene promotors and RNA, as well as in telomeric DNA (10–12), which are central for a functioning DNA metabolism. Thus, G-quadruplexes play a role in a number of biological processes and have implications for human health (13,14). Many places in the genome, including the ribosomal DNA, the immunoglobulin heavy-chain switch regions, some promotors and the telomeres, have the potential to fold multiple consecutive G-quadruplexes, where two or more G-quadruplex units form simultaneously (15–24). The G-quadruplex–G-quadruplex interface has therefore been suggested as a new potential drug target (25–29). We focus here on human telomeric DNA, which contains the repeating TTAGGG sequence. Telomeres terminate in an ∼200-nucleotide-long single-stranded 3′ DNA overhang, thus enabling the potential formation of several consecutive G-quadruplexes.

G-quadruplexes are made by the stacking of guanine quartets and stabilized by monovalent cations, such as K^+^ and Na^+^. Several stable G-quadruplex conformations, defined by backbone orientation and internal loop type, have been identified for the human telomeric sequence (30–35). The prevalence of specific G-quadruplex conformations is defined by factors such as ion type and concentration, solvent composition, and length and sequence of loops and flanking regions of the G-quadruplex-forming sequence (36,37). For telomeric DNA, NaCl leads to primarily antiparallel conformations, whereas in KCl hybrid conformations (hybrid 1 and hybrid 2) as well as antiparallel and parallel conformations have been reported (33,38). Telomeric sequences with >4 GGG repeats can support G-quadruplex formation at different positions and eventually multiple G-quadruplexes can form if the repeat number is ≥8 (39,40). A single-molecule tweezers study showed that random formation of G-quadruplexes in long human telomere overhangs leads to a (fast) kinetic folding pattern with targetable vacant G-tracts (41). Recent investigations proposed that vacant G-tracts are also present in equilibrium conditions (42–44). However, multiple scientific investigations have shown that consecutive (close-packed) G-quadruplexes form under equilibrium conditions (28,45–50). Studying the G-quadruplex’s thermal stability in long human telomeric DNA strands (able to support one to three consecutive G-quadruplex units) revealed that flanking nucleotides influence G-quadruplex stability so that terminally positioned G-quadruplexes are thermodynamically more stable than internally positioned G-quadruplexes (49,51). *In vitro* studies of consecutive G-quadruplexes have indicated that interactions between telomeric G-quadruplex units are minor (49), supporting the theory that consecutive G-quadruplexes largely act as non-interacting *beads on a string* (45,48). Nevertheless, several studies indicated that negative cooperativity may play some role in the folding of consecutive telomeric G-quadruplexes under physiologically relevant conditions and prevent their close packing (43,47). To further complicate matters, significant positive cooperation between antiparallel G-quadruplex units has been reported under special conditions leading to stable consecutive G-quadruplex folding (50).

Folding of one G-quadruplex was found to follow a kinetic partitioning model process (52,53). Single-molecule FRET approaches have contributed with a direct view of the dynamics and conformational heterogeneity of a G-quadruplex (54–60). Here, we used single-molecule FRET in combination with circular dichroism (CD), thermal melting and molecular dynamics (MD) simulations to investigate conformational heterogeneity and folding dynamics of consecutive G-quadruplexes in human telomeric DNA sequences with experiments bridging both nonequilibrium and equilibrium conditions. Our results show that the folding process with an eight-repeat telomeric DNA construct results in a complex situation with multiple conformational populations present at equilibrium, including both fully and partially folded structures. In buffer conditions that promote G-quadruplex formation (100 mM K^+^ or Na^+^), the fully folded conformation (two consecutive G-quadruplexes) was the dominant population. However, there was a significant persisting population of partially folded structures. We find that our experimental data are well reproduced by an *off-path folding model*, where multiple G-quadruplex conformations, forming in the middle of the sequence, block the formation of tightly packed consecutive G-quadruplexes, thus explaining the persistent partially folded population.

## MATERIALS AND METHODS

### Oligonucleotides and chemicals

All salts and chemicals were purchased from Sigma–Aldrich (Germany), unless otherwise specified. Fluorescently labeled DNA oligonucleotides were purchased from IBA (Germany) and HPLC or PAGE purified by the manufacturer. Oligonucleotides used for single-molecule analysis contained a biotin at the 5′ end used for surface immobilization. Unlabeled oligonucleotides were purchased from Sigma–Aldrich and HPLC purified by the manufacturer.

### Sample preparation

G-quadruplex-forming oligonucleotides (Supplementary Table S1) were dissolved in annealing buffer (20 mM Tris–HCl, pH 7.5, with KCl, NaCl or LiCl in the noted concentration). The sample was thermally annealed in a water bath, by incubating it at 95°C for 5 min, followed by gradually cooling to room temperature over 16 h.

### Single-molecule FRET experiments and data analysis

Single-molecule FRET experiments were performed on surface-immobilized DNA molecules using a prism-based total internal reflection fluorescence microscope (based on a Zeiss microscope). Double-labeled DNA molecules (∼2.5 pM) were immobilized inside a cover slide chamber (a pair of quartz/glass slides assembled by Parafilm stripes) using BSA–biotin and streptavidin anchoring. The excess of non-immobilized labeled molecules was washed out by flushing the chamber with excess dilution buffer (20 mM Tris–HCl, pH 7.5, with KCl, NaCl or LiCl in the noted concentration). Detailed protocols of these experimental procedures can be found in ref. (61). Prior to imaging, the cover slide chamber was flushed with an imaging buffer consisting of the dilution buffer supplemented with an oxygen scavenging system composed of Trolox (2 mM), glucose oxidase (17 U ml^−1^), catalase (260 U ml^−1^) and glucose (4.5 mg ml^−1^). Fresh imaging buffer was flushed into the chamber every 20 min, to avoid pH gradients. The excitation of fluorophores was achieved using an alternating laser scheme (62) with 532 and 640 nm lasers (Cobolt), respectively. Fluorescence from the donor and acceptor fluorophores was spatially separated onto the EMCCD camera (Andor, iXON 3) by a wedge mirror (Chroma). Movies were recorded with a 200 ms integration time per frame with a total length of 500 s (8.3 min). Several independent samples were measured for each buffer condition. Data analysis was performed using the iSMS software (63) running in MatLab version 2016b and 2018b (MathWorks). The FRET efficiencies were obtained from the donor and acceptor fluorescence intensities as follows:

$$\begin{equation*}E = \frac{{{F_{{\rm A}|{\rm D}}}}}{{{F_{{\rm A}|{\rm D}}} + \gamma {F_{{\rm D}|{\rm D}}}}},\end{equation*}$$

where *F*_A|D_ and *F*_D|D_ denote the donor and acceptor fluorescence intensities after donor excitation, respectively, that were corrected for background signal, donor leakage (*α* = 0.15) and acceptor direct excitation (*δ* = 0.05) contributions. The *γ*-factor (*γ* = 1.2) was used to account for differences in brightness and detection efficiency between the donor and acceptor fluorophores (64). The correction factors (*α*, *δ* and *γ*) were determined for all samples based on single-molecule fluorescence time-traces. Average values were used for correcting the transfer efficiencies for all datasets. Only fluorescence time-traces with single-step donor and/or acceptor photobleaching were selected for further analysis and used to build single-molecule FRET histograms (Supplementary Table S2). Each frame in a time-trace gave one count in the single-molecule FRET histograms. Single-molecule FRET trajectories showing conformational dynamics were analyzed using hidden Markov modeling (HMM) with the variational Bayesian expectation maximization technique (63,65). The obtained lifetimes were plotted in histograms and fitted with a simple exponential decay function (OriginLab, 2016). To estimate error bars on fractions of FRET populations, the pool of FRET trajectories at each condition was randomly split into three equal parts and FRET histograms were built based on each of these data subsets, yielding three histograms per condition. Population fractions were determined for each of these histograms and averaged. The folding mechanism of the eight-repeat telomeric DNA construct (Tel8) was modeled in MatLab through rate equations for each of the states in the system.

### FRET melting

FRET spectroscopy measurements were carried out with a FluoroMax-3 fluorimeter by Horriba Yvon with a coupled Wavelength Electronics LFI-3751 Peltier element for temperature control. Quartz cuvettes with a 3 mm light path were used. The slit width was set to 5 nm and the detection integration time to 0.1 s. The donor fluorophore (Cy3) was excited at 530 nm; fluorescence intensity was measured between 540 and 720 nm. The acceptor fluorophore was excited at 600 nm; the emission was measured between 610 and 720 nm. FRET efficiency was obtained by measuring the emission spectra upon donor excitation. From these spectra, the FRET efficiency was determined by dividing the acceptor peak fluorescence intensity (*I*_DA_) by the sum of donor and acceptor peak intensities, *I*_DD_ and *I*_DA_, respectively:

$$\begin{equation*}E = \frac{{{I_{{\rm DA}}}}}{{\left( {{I_{{\rm DA}}} + {I_{{\rm DD}}}} \right)}}.\end{equation*}$$

### UV/Vis absorbance and CD spectroscopy

Unlabeled and labeled oligonucleotides were prepared in 50 mM Tris–HCl, pH 7.5, with appropriate salt as stated in the text, and had a final DNA concentration of 2.5 μM. UV or CD spectra were obtained using a UV/Vis spectrophotometer (PerkinElmer Lambda 25) or a CD spectrophotometer (Jasco J-810) measuring the sample in a 1 cm path length 3 ml or 100 μl quartz cuvette (Hellma), respectively. All melting curves were acquired at 295 nm with a gradient of 0.2°C min^−1^. Melting curves were plotted as described in ref. (66). CD spectra were recorded at room temperature unless otherwise specified and background corrected before analysis.

### Molecular modeling

Atomistic and coarse-grained (CG) MD simulations were performed for atomistic models of tandem G-quadruplex constructs (see Supplementary Table S3) containing several important G-quadruplex conformations and consisting of two hybrids (PDB 2JSM), two baskets (PDB 143D), two chairs (PDB 2KM3 was used as a template) and mixed basket–chair G-quadruplex conformations. The models were then ‘trimmed’ at both ends and have the same sequence for all models: 5′-(TTAGGG)_8_-TT-3′. Systems were solvated in a dodecahedral box of size (8.92 × 8.92 × 6.3) nm^3^ containing 15 980 TIP3P (67,68) water molecules and 200 mM NaCl (‘basket’ and ‘chair’ folds) or KCl (‘hybrid-1’ folds). Ion parameters derived by Joung and Cheatham were used (69), and for DNA the Amberff-OL15 force fields including ϵ/ζOL1 (70), χOL4 (71) and βOL4 (72) modes for Amber-ff14 were used.

The systems were equilibrated with the ‘Berendsen’ barostat (1 atm, *τ* = 2.0 ps^−1^) and thermostat (*τ* = 1.0 ps^−1^) with a temperature ramp from 10 K to the final simulation temperature of 300 K in six steps (100 ps each step, integration step 1.0 fs) applying Cartesian constraints to the DNA atoms (starting at 1.0 kJ mol^−1^ nm^−2^). The Cartesian restraints on the DNA were slowly reduced and each system was finally equilibrated in the NPT ensemble using the ‘Parrinello–Rahman’ barostat and the ‘v-rescale’ thermostat. Hydrogen covalent bonds were constrained to their equilibrium length allowing for an integration timestep of 2 fs. Simulations were performed using the Gromacs v5.1.4 software (73). The resulting trajectories were stored on disk every 10 ps for subsequent analysis.

#### Coarse-grained force field

The atomistic G-quadruplex tandem models were converted to CG models using an in-house modified version of the ‘martinize.py’ script, and a slightly modified version of the MARTINI-DNA force field (74), as original interactions were parameterized for standard B-DNA and did not allow for stable insertion of ions between the G-quadruplex quartets (see Supplementary Figure S1 and Supplementary Tables S3–S5). The CG systems were equilibrated at 310 K and simulated using Gromacs v5.1 software. The resulting trajectories were stored on disk every 100 ps for subsequent analysis (see Supplementary Table S3).

#### Analysis

The MD simulations were analyzed with in-house Python scripts using the MDAnalysis package and GROMACS tools. The most stable structures explored by the systems were identified using the gromos clustering algorithm implemented in gmx cluster (75). The cutoff for each system was determined by looking at the position of the first peak in the probability distribution of RMSD between each pair of structures. An RMSD cutoff of 0.7 or 0.1 nm was used for the CG-MD (full G-quadruplex structure) and atomistic MD simulations (interface loops only), respectively. The choice of cutoffs was made by visual inspection of the position of the first peak in the RMSD distribution between each simulation frame used in the clustering. Accessible volume calculations were performed using the FSP software v1.1 (76), assuming fluorophores on the last base on each side of the oligo. Conversion from distance to approximate FRET values was determined using *R*_0_ = 52 Å.

## RESULTS

### Populations with various degrees of DNA folding coexist in human telomeric DNA sequences in equilibrium conditions

**Figure 1.**
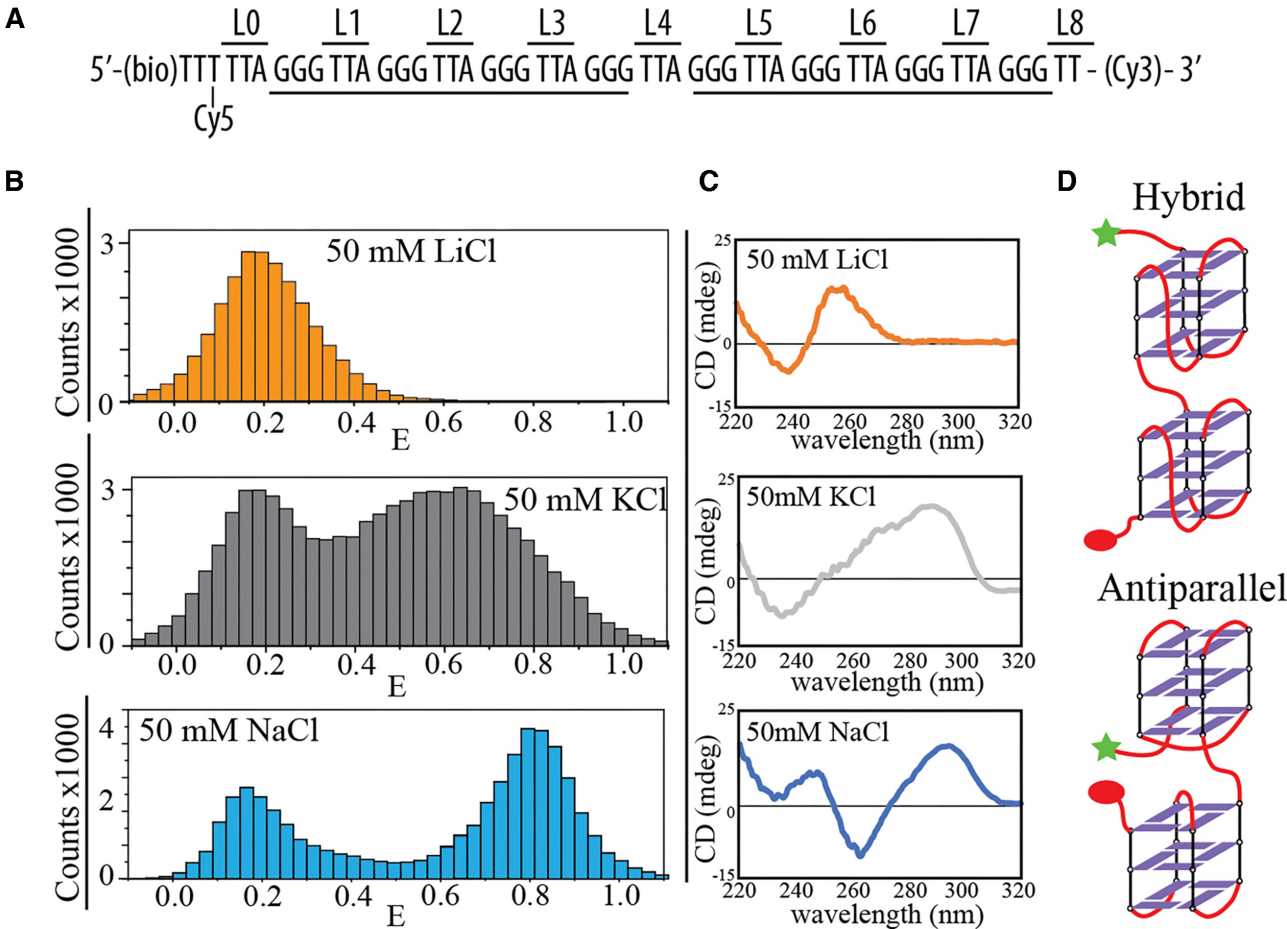
Dominant DNA conformations and conformational distributions of the telomeric repeat construct (Tel8). (**A**) Nucleotide sequence of Tel8 with positions of cyanine dyes (Cy3 and Cy5) and biotin (bio) labeling. L0–8 define the TTA linker position relative to the 5′ end of the DNA construct. (**B**) FRET histograms of single-molecule data from single-molecule analysis of Tel8 in the presence of 50 mM LiCl, KCl or NaCl. (**C**) CD spectra of unlabeled Tel8 in the presence of 50 mM LiCl, KCl or NaCl. (**D**) Schematic drawing of consecutive G-quadruplexes, either in a hybrid–hybrid conformation or in a basket–basket conformation (lower). Star and sphere indicate positions of Cy3 and Cy5 fluorophores, respectively.

As a model for multiple telomeric G-quadruplex folding that can fold two consecutive G-quadruplexes, we investigated a dye-labeled construct containing eight human telomeric (TTAGGG) repeats, termed Tel8 (Figure 1A), which can fold two consecutive G-quadruplexes. Single-molecule FRET measurements were performed in thermodynamic equilibrium conditions after sample annealing (see the ‘Materials and Methods’ section). A variety of transfer efficiency populations were observed in the presence of KCl, NaCl and LiCl (Figure 1B). FRET melting experiments showed clear melting transitions in the presence of KCl and NaCl but not LiCl (Supplementary Figure S2). Lithium ions do not promote G-quadruplex formation (77). Mostly unfolded DNA was observed in these conditions as further evidenced by low FRET efficiencies (E) with E ∼ 0.2 (Figure 1B) and by the lack of G-quadruplex signatures in the CD spectra (77,78) (Figure 1C). In contrast, G-quadruplex formation was favored in the presence of potassium and sodium ions as evidenced by the presence of high FRET efficiency populations (Figure 1B) and strong CD signals, indicative of multiple G-quadruplex formation (Figure 1C and Supplementary Figure S2). FRET population distributions were different in the presence of KCl and NaCl showing the presence of different folded DNA conformations for the two salts. CD analysis can be used to identify the nature of the conformations observed in NaCl and KCl (78). CD measurements of Tel8 showed characteristic hybrid signature (CD peaks at 290 and 275 nm, and valley at 235 nm) in the presence of KCl and antiparallel signature (CD peak at 295 nm and valley at 265 nm) in the presence of NaCl (Figure 1C). Both unlabeled and labeled constructs showed very similar CD features in the presence of NaCl. However, the CD curves were affected by the labeling in the presence of KCl (Supplementary Figure S2). A close look at the G-quadruplex conformations comparing consecutive hybrid–hybrid and antiparallel–antiparallel G-quadruplexes shows that the orientation of overhangs in the latter leads to closer proximity for the dyes (Figure 1D). Therefore, higher transfer efficiencies are expected for consecutive antiparallel G-quadruplexes. These structural considerations fit well with the observation of a higher transfer efficiency population in the presence of NaCl at *E∼* 0.8 (assigned to primarily antiparallel conformations) (Figure 1B).

The broad FRET distributions with multiple peaks in the presence of both KCl and NaCl, containing both low and high FRET states (Figure 1 and Supplementary Figure S3), imply the coexistence of several conformations in equilibrium conditions with only part of the population containing two G-quadruplexes.

### Low FRET states encompass populations with different DNA foldings that can form one G-quadruplex

**Figure 2.**
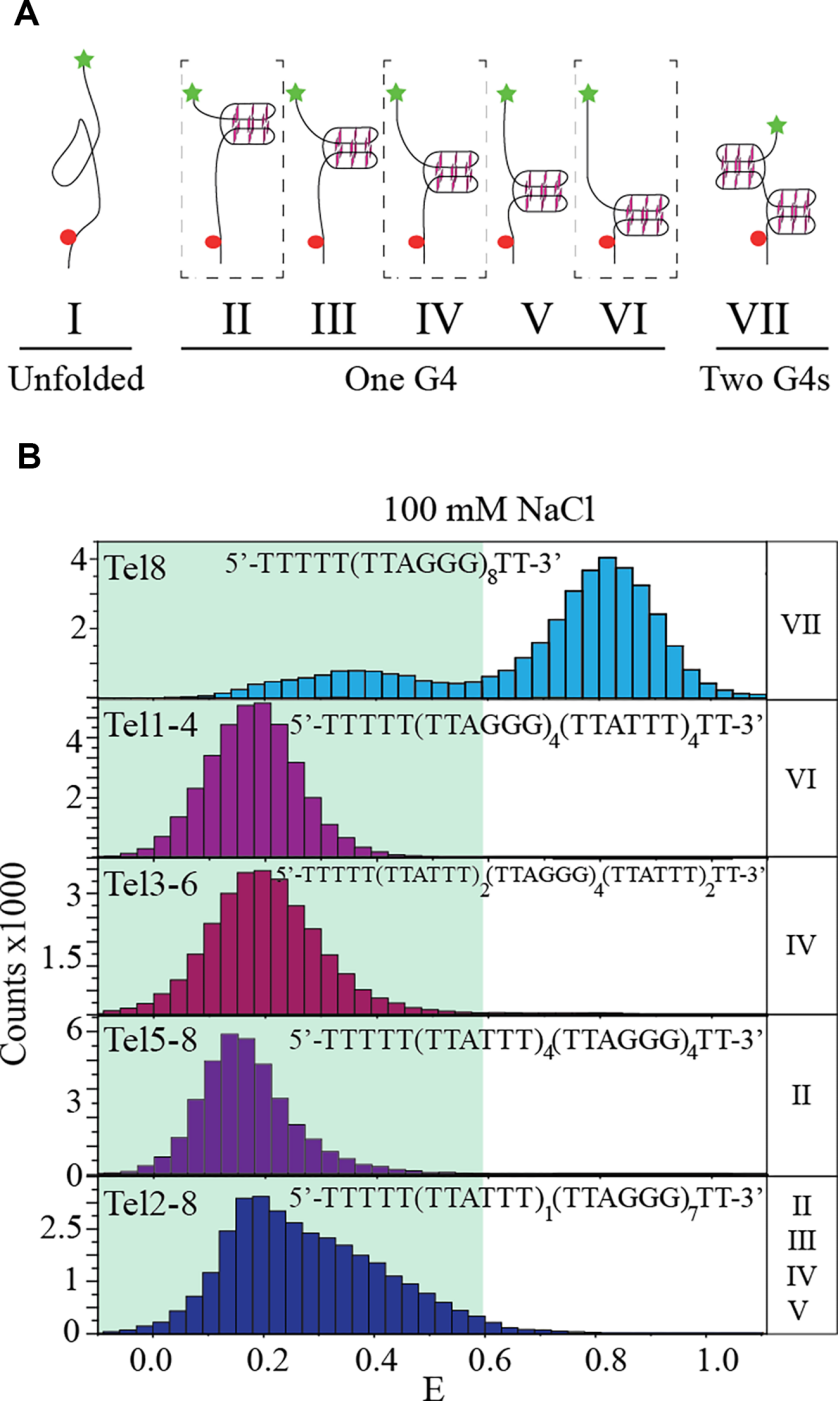
Identification of the population of conformations observed in Tel8 in the presence of 100 mM NaCl. (**A**) Cartoon drawings of possible G-quadruplex (G4) folding in Tel8 including no G-quadruplexes (unfolded), one G-quadruplex at several positions or two G-quadruplexes. In the drawings, G-quadruplexes are illustrated by three stacking guanine tetrads connected by backbone DNA (black lines). Stars and spheres show Cy5 and Cy3 positions, respectively. (**B**) FRET histograms of single-molecule data of Tel8 or mutant versions of Tel8. The shaded area in the histograms marks *E*< 0.6. The name of mutants is noted at the top of their respective histograms. The roman numbers to the right of each histogram refer to the conformations illustrated in panel (A) where G-quadruplexes are expected to form.

Telomeric constructs of eight TTAGGG repeats can take many different conformations, including unfolded conformations, one G-quadruplex structure at different positions in the construct or two G-quadruplexes (40). Those conformations (termed I–VII) are illustrated in Figure 2A. To analyze G-quadruplex formation at specific positions along the DNA construct, we designed a set of Tel8 mutants, Tel1–4, Tel3–6, Tel5–8 or Tel2–8, where GGG repeats outside noted positions were mutated to TTT (see the primary sequence of mutants in Supplementary Table S1). Tel1–4 can thus fold one G-quadruplex with the first four repeats from the 3′ end (conformation II in Figure 2A), Tel3–6 with the middle four repeats (conformation IV) and Tel5–8 with the last four repeats (conformation VI). Tel2–8 only had the first GGG repeat mutated and can have one G-quadruplex at various positions using repeats 2–8 (conformations II–VI). All the mutants can form only one G-quadruplex. CD spectroscopy and thermal melting investigations were used to validate G-quadruplex formation of the mutants in the presence of 100 mM NaCl and KCl. All mutants showed melting, thermodynamic parameters and CD signals consistent with the formation of one G-quadruplex (Supplementary Figure S4). G-quadruplex formation was not observed in 100 mM LiCl (Figure 1 and Supplementary Figure S2). Single-molecule FRET histograms for each of the mutants under folding conditions (100 mM NaCl or KCl) and unfolded conditions (100 mM LiCl) showed predominantly low to intermediate transfer efficiencies (*E* < 0.6) (Figure 2B and Supplementary Figure S5). Thus, the low FRET populations observed for Tel8 (Figure 1B, NaCl and KCl histograms) represent not only unfolded but also partly folded conformations, including conformations with one G-quadruplex (II–VI, Figure 2). Possibly other conformation, such as G-hairpins and G-triplexes (79–81), may also be present.

### Single-molecule dynamics show transitions between the different FRET states

**Figure 3.**
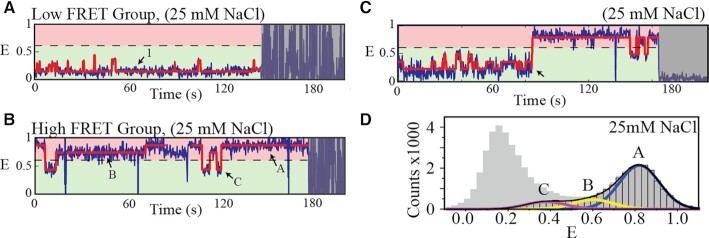
Analysis of single-molecule FRET time-traces. Results of HMM fitting are shown by red lines. (**A**, **B**) Representative FRET time-traces from the two main group classifications, respectively. Dashed line marks *E*= 0.6. Specific FRET states corresponding to mostly unfolded (1) as well as partially and fully folded DNA conformations (A, B and C) are marked by arrows. (**C**) Representative FRET time-trace of the small population of the high FRET group that features transitions from the lowest to the highest FRET state (event marked by a black arrow). (**D**) FRET histogram showing single-molecule data from the low (gray with no edge) and high (gray with black edge) FRET groups.

Single-molecule time-trace measurements of Tel8 showed many transitions between FRET states during the observation time window (in the presence of KCl and NaCl) (Figure 3 and Supplementary Figures S6 and S7), indicating that many of the observed DNA conformations (but not all) were metastable. In conditions where G-quadruplex folding was not favored (in the presence of LiCl), FRET histograms showed only one main low FRET peak and very little dynamics further indicating that the DNA was mostly unstructured in these conditions (82) (Supplementary Figures S3 and S6).

We focused on the NaCl situation, which offers conditions where the low and high FRET populations (unfolded/partially folded and fully folded populations, respectively) were well separated (Figures 1 and 2). FRET time-traces showed many transitions (Figure 3 and Supplementary Figure S7; the latter shows data for different NaCl concentrations). All FRET time-traces could be grouped into two categories for further analysis: (i) a low FRET group, where FRET time-traces only contained states with *E* < 0.6 (Figure 3A), and (ii) a high FRET group, where states with *E* ≥ 0.6 were also present (Figure 3B and C). The fully folded conformations thus are only present in the high FRET group (Figures 2 and 3).

Within the low FRET group, time-traces showed transitions from a distinct lowest FRET state (termed state 1) to multiple slightly higher (intermediate) FRET states (Figure 3A). HMM analysis showed that the lifetime of state 1 decreased with increasing salt concentration (Supplementary Figure S8). At the same time, the FRET efficiency of state 1 (the lowest FRET state) increased (Supplementary Figure S7). These features match well with what would be expected from an unfolded DNA single strand (82). FRET efficiencies of the remaining states (*E* ∼ 0.2–0.5) match with expectations from partly folded conformations, including those of one G-quadruplex (conformations III–V in Figure 2A). This analysis allowed identifying the unfolded population as the lowest FRET state in each time-trace in the low FRET group, even though this identification was not possible looking only at the histograms (Figure 2 and Supplementary Figure S5).

Within the high FRET group, we identified three FRET states (A, B and C) (Figure 3B). States A (*E* ∼ 0.8) and B (*E* ∼ 0.6) had long lifetimes and are thus assigned to different fully folded conformations. States B were much less populated making states A the dominant fully folded population (Figure 3D). States C (*E* ∼ 0.4) reached down to the partly folded population (with FRET efficiencies similar to those observed in the low FRET group). States C had much shorter lifetimes than the fully folded conformations, and direct transitions to states A and B. Thus, states C are assigned to partly unfolded conformations of states A and B. Full lifetime analysis of these states is shown in Supplementary Figure S8.

A low fraction (∼5%) of the high FRET group time-traces also showed very low FRET states (*E* ∼ 0.2). These rare events (arrow in Figure 3C) were associated with transitions between the unfolded or partly folded and the fully folded DNA (Supplementary Figure S9). Interestingly, transitions between low and high FRET states were frequent in the presence of KCl (Supplementary Figure S6, top panel).

The rarity of direct transitions between the lowest and highest FRET states in NaCl conditions and the observed FRET dynamics point to a folding mechanism having several folding pathways and where only a subset of those (the on-path conformations) lead to full folding into two G-quadruplexes.

### Time-course investigation reveals the kinetics of full folding

**Figure 4.**
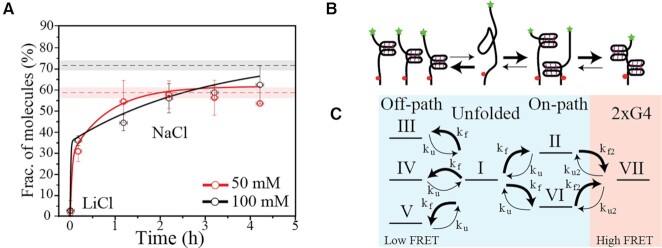
Time-course investigation of Tel8 condensation. (**A**) Fraction of fully folded DNA (*E*≥ 0.6) as a function of time after buffer change from LiCl to NaCl. Data from single-molecule FRET time-course experiments in the presence of 50 and 100 mM NaCl are shown as red and black circles, respectively. Red and black dashed lines mark the proportion of fully folded molecules measured under equilibrium conditions (Supplementary Figure S3); the uncertainty is shown as transparent shading. Red and black lines represent modeled fractions of fully folded G-quadruplexes using experimentally determined reaction rates from single-molecule FRET time-traces taken with 50 and 100 mM NaCl, respectively (see Supplementary Table S6 and Supplementary Figure S13). (**B**) Simple cartoon of the proposed folding mechanism. Folded structures are shown as G-quadruplexes, but other type of structures may be present in the off-path branch. Initial folding leads to the formation of both fully folded states and off-path structures. Over time the off-path structures collapse and allow refolding to reach the fully folded state of two G-quadruplexes. (**C**) Detailed model for the proposed folding mechanism, including unfolded, off-path, on-path and two G-quadruplexes (2xG4) conformations, which yielded the full lines in panel (A). Roman numbers refer to the conformations illustrated in Figure 2A. The thickness of the reaction arrows in panels (B) and (C) indicates the expected relative size of the rate constants for either folding or unfolding (*k*_f_ or *k*_u_, respectively) of the one G-quadruplex or folding or unfolding of the second G-quadruplex (*k*_f2_ or *k*_u2_, respectively).

Conditions where partially and fully folded structures coexist are determined by folding pathways and dynamics. To further investigate the folding process and mechanism of consecutive G-quadruplex formation, we performed time-course single-molecule FRET experiments with Tel8 in NaCl buffers. This approach allowed detecting the folding kinetics of the fully folded structures (defined as *E* ≥ 0.6) over the course of several hours (Figure 4A). Tel8 was initially prepared in a LiCl-containing buffer preventing G-quadruplex formation (plotted as time zero in Figure 4A). Buffer conditions were then rapidly changed by replacing the solution in the reaction chamber with a buffer containing 50 or 100 mM NaCl to trigger G-quadruplex formation. Single-molecule trajectories were clustered into histograms around the noted time points (Supplementary Figure S10) and the proportion of fully folded molecules for each histogram was plotted as a function of time after buffer exchange (Figure 4A). Already at the first measuring point, only a few minutes after folding initiation, we observed that a fraction of the population was fully folded (Figure 4A). During the following ∼4 h, this fraction increased slowly toward equilibrium values. We note that the time to reach equilibration was slower at 100 mM than at 50 mM NaCl. This difference in equilibration time between the two buffer conditions is also observed with CD experiments (Supplementary Figure S11). Slower equilibration at higher NaCl concentration is surprising since the folding rate of one G-quadruplex increases with salt (58,83).

Based on the observed equilibrium dynamics and folding kinetics, we propose a folding mechanism (the off-path folding model), illustrated in Figure 4B, where G-quadruplex structures initially form at multiple positions along Tel8 (conformations II–VI in Figure 2A) and are later converted to fully folded conformations. A more detailed description of the model is illustrated in Figure 4C. The conversion to fully folded conformations is limited by the collapse of off-path conformations (such as conformations III–V in Figure 2). According to this model, higher salt would stabilize these off-path G-quadruplex conformations, thus leading to slower equilibration at high NaCl, as seen in Figure 4A. Only the on-path conformations (II and VI, 40%) allow the direct formation of two G-quadruplexes (conformation VII), whereas the off-path conformations (III–V, 60%) first need unfolding. Assuming kinetic equipartitioning and equal probability of G-quadruplex formation (positions II–VI), we expect 40% of the population to be on-path for initial full folding. Indeed, we saw that 34 ± 12.5% and 36 ± 6.1% were fully folded at the first time point after folding, at 50 and 100 mM NaCl, respectively (Figure 4A), indicating that initial G-quadruplex formation was largely randomly distributed within the Tel8 sequence.

Our model described the experimental single-molecule time-course data well, when simply assuming one unfolding and one folding rate, i.e. equal rate of folding and unfolding (equal stability) for all G-quadruplexes independently of position (Supplementary Figure S12). The best fit parameters adequately showed an increase in the Gibbs free energy (Δ*G*) of G-quadruplex folding with increasing salt concentration, which compared reasonably well with our thermal melting results (Supplementary Figure S4E). However, this simple model did not catch the full physical realty of the folding process. Indeed, the fit parameters showed an increased lifetime of the unfolded state with increasing salt concentration, which is not physically expected and does not match with experimental observations (see the previous section).

Using experimentally determined parameters from our single-molecule investigations in NaCl (Supplementary Figures S8 and S9), we determined the rate constants *k*_u_, *k*_f_, *k*_u2_ and *k*_f2_, unfolding and folding rates for the first and second G-quadruplexes, respectively (Supplementary Table S6), and directly used the obtained values in our model. This approach reproduced the observed time-course behavior well despite the experimental uncertainties, especially for *k*_u_ (Figure 4A and Supplementary Figure S13). The G-quadruplex folding rates increased with increasing salt concentration, which is physically expected. We observe that the rate constant for the folding of the second G-quadruplex (*k*_f2_) from state C was larger than that for folding of the first G-quadruplex (*k*_f_) from state 1, which could imply that the first folded G-quadruplex, when in an on-path conformation, can template folding of the second G-quadruplex. Positive cooperativity has previously been reported for consecutive telomeric G-quadruplexes in sodium (50).

Taken together, our data catch a direct view of the DNA folding process at both short and longer times, i.e. in nonequilibrium and equilibrium conditions. Our model proposes that G-quadruplexes can fold at many positions along the DNA, thus affecting DNA global folding.

### *In silico* modeling of tandem G-quadruplex conformations

**Figure 5.**
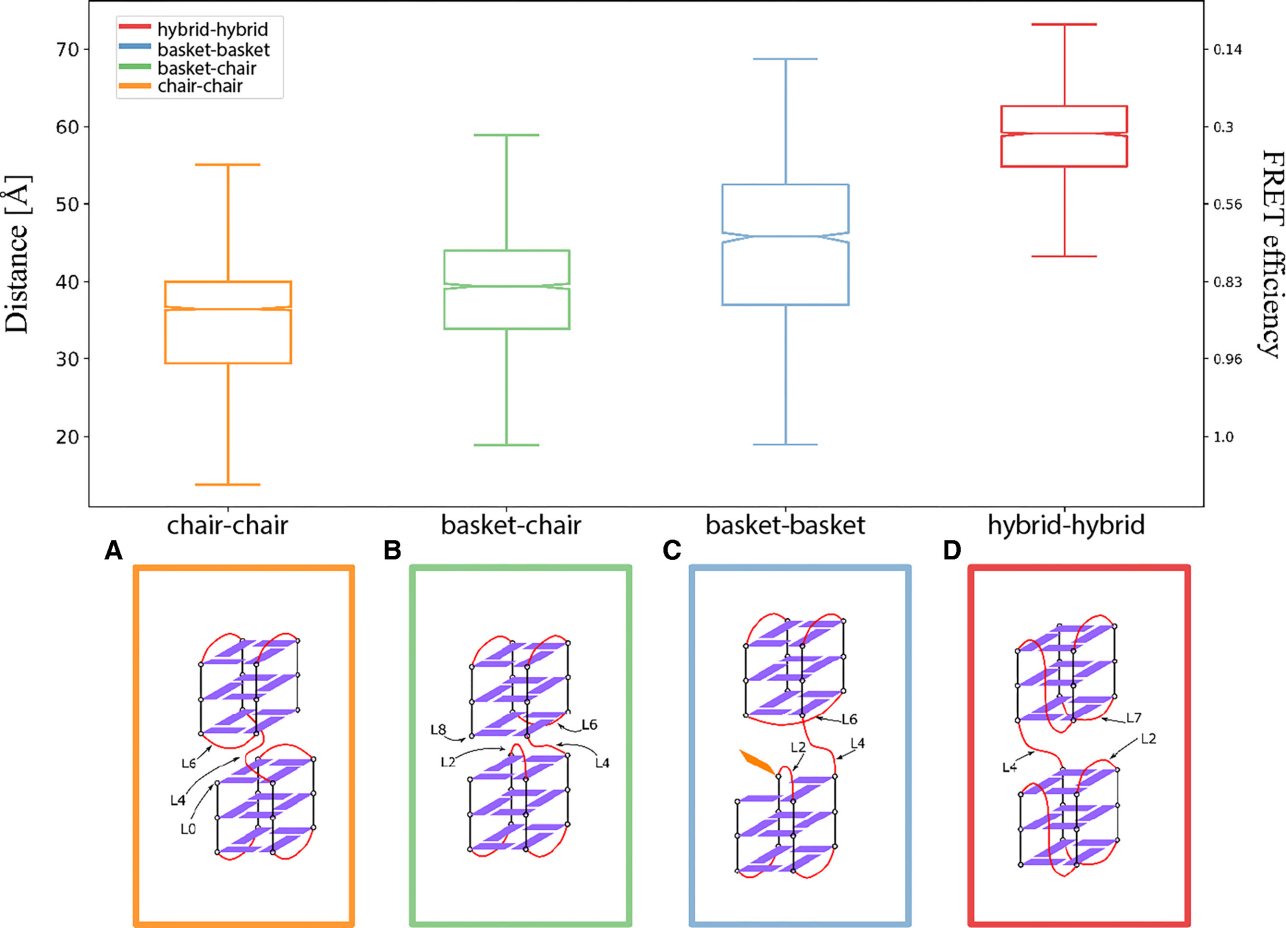
Summary of results from G-quadruplex *in silico* CG modeling. Top panel: Box plot of the accessible volume average distances (in Å, left-side *y*-axis) and the resulting FRET (right-side *y*-axis) calculated using a Förster radius of 52 Å. The box contains the central interquartile of the sample (central 50% around the median), the bars extend 1.5 times the interquartile range, the line indicates the sample median and the notch is the median 95% confidence interval. Bottom panel: Schematic representations for all CG models: (**A**) chair–chair system; (**B**) mixed basket–chair system; (**C**) basket–basket system; (**D**) hybrid–hybrid system. Guanine bases are represented as a violet rectangle when forming a G-quartet or as orange rectangle otherwise. Connecting loops are represented as red curved lines. The loops that are observed forming relevant interaction at the interface between the G-quadruplexes are labeled using the naming scheme reported in Figure 1.

Several high FRET states were observed in our single-molecule analysis in potassium and sodium representing different conformational variants of two G-quadruplexes. To have a closer look at some of the highly folded conformations present in the eight-repeat Tel8 construct, we modeled four different G-quadruplex conformational combinations potentially present (hybrid–hybrid, basket–basket, basket–chair and chair–chair) using CG models (see Supplementary Table S3). CG simulations were performed to maximize the relative G-quadruplex orientation’s sampling (microseconds). The centroid of the two most populated conformation clusters observed in the CG-MD (corresponding to the two most probable dye–dye distances) was then back-mapped to all-atom (AA) conformations and used as starting point for fully AA-MD simulations. Compared to CG-MD, AA-MD simulations provided a better method for investigating precise molecular interactions and allowed investigations of the relative positioning of the dyes (attached to the DNA construct) and interaction patterns between elements in the analyzed G-quadruplex conformations. We obtained large distance distributions for each investigated construct, indicative of movement of the two G-quadruplex structures on the fast timescale sampled by simulations (Supplementary Figure S14). Our experimental observations were done on a much longer timescale and cannot resolve the fast dynamics observed in simulations. To obtain information on average conformations, we determined average dye–dye distances from the simulation data (Figure 5). Based on that we see that the hybrid–hybrid conformation had on average the largest dye–dye distance followed by the basket–basket, basket–chair and chair–chair conformations (Figure 5 and Supplementary Table S7).

Contact analysis of the clusters (see Supplementary Figures S15–S17) showed that the two tandem G-quadruplex units interact mainly via loop interactions. In all systems, the connecting loop (L4) was involved at the interface between the two G-quadruplex folds [see loop positions (L0–L8) in the construct schematically illustrated in Figure 1A. In the hybrid–hybrid system, the more probable contact was between L4 and L7, while in the basket–basket, chair–chair and basket–chair systems, L4 was found more often at contact distance with L6. In the chair–chair system, detectable contacts between L6 and the 5′-terminal (L0) were observed. The most stable conformations explored during the AA-MD simulations are described in Supplementary Figures S14 and S15. In all systems, except for the basket system, base stacking between G-quartets from each of the two G-quadruplexes and loop bases was observed.

In general, the *in silico* modeling indicates that the analyzed tandem G-quadruplex conformations impose geometrical restrains resulting in distinct dye–dye distance patterns. We see a small effect of inter-G-quadruplex interactions between consecutive folds suggesting a possible minor cooperative effect when both G-quadruplexes have formed. The simulations show that the basket–basket configuration is least compatible with the formation of stable higher order structures. This is due to the two ‘crossing’ loops at the interface between the two G-quadruplexes that result in a disturbance of the stable interactions. The crossing loops also result in the broader dye–dye distance distribution observed for the basket–basket conformation (blue histogram in Supplementary Figure S14, top panel).

## DISCUSSION

Multiple sites in the human genome have G-rich DNA stretches able to support consecutive G-quadruplex formation, one of which is the telomeric overhang region. The repetitive nature of telomeric DNA implies that G-quadruplexes can fold at several places along the DNA, which complicates the folding landscape (24,40,41,43,44,84). We investigated the folding of DNA containing human telomeric repeats, supporting the formation of up to two consecutive G-quadruplexes, over the seconds to hours timescales to elucidate the importance of alternative conformations on the formation of consecutive G-quadruplexes. Our results, bridging nonequilibrium and equilibrium conditions, revealed that long telomeric overhang sequences give rise to large conformational heterogeneity, with the coexistence of multiple conformational dynamic states at equilibrium. This in turn led to noncomplete DNA folding as observed in the presence of both KCl and NaCl and described by our off-path folding model.

Folding of consecutive G-quadruplexes in NaCl buffers showed noncomplete DNA condensation (Figure 4 and Supplementary Figures S3 and S11) and is a clear case of frustrated folding. G-quadruplexes appeared to nucleate randomly in our eight telomeric repeat sequence resulting in an *off-path* and an *on-path* population of conformations containing one G-quadruplex, where only the *on-path* population allows the formation of the second G-quadruplex (Figure 4). The fully folded conformations showed high FRET efficiencies and CD spectra indicative of antiparallel conformations (Figures 1 and 3D and Supplementary Figures S2 and S11) and could be represented by the chair–chair, chair–basket and basket–basket (antiparallel) constructs modeled in MD simulations (Figure 5). The basket–basket construct was least compatible with the formation of stable higher order structures in part due to the collision of the L2 and L6 linkers, which is likely to increase with higher salt due to more backbone charge screening.

In the single-molecule data, we saw in NaCl buffers a very low number of transition events from the lowest to the highest FRET state, i.e. from unfolded/partly folded to fully folded DNA (Figure 3C). Slow kinetic conformational equilibration was observed with both single-molecule FRET and CD spectroscopy (Figure 4 and Supplementary Figure S11). This together is indicative of a strong energy barrier for low/high FRET transitions. Importantly, this energy barrier appears to be a result of very slow unfolding in NaCl buffer (low *k*_u_ and *k*_u2_; see Supplementary Table S6) and not of slow folding of the second G-quadruplex (as we see a high *k*_f2_). We speculate that this transition barrier may reflect that the conformational states that can be visited in NaCl (mostly antiparallel) force the DNA to significantly unfold in order to interconvert between off-path and on-path conformations.

In KCl buffers, folding of consecutive G-quadruplexes has recently been described both to be dominated by the fully folded lowest free energy conformations (28) and to follow a frustrated folding model (43). In this study, we observed many overlapping states in potassium buffers (Supplementary Figures S3 and S6). Although a detailed quantification of these states was not performed, mainly because folding appeared affected by interactions with fluorophores (Supplementary Figure S2), we overall saw a similar pattern to that in NaCl of unfolded and partly unfolded conformations (low FRET) and fully folded two G-quadruplexes (intermediate to high FRET) (Figure 1 and Supplementary Figures S3 and S5). The FRET efficiency of the fully folded two G-quadruplexes in potassium and CD data fit well with the hybrid–hybrid conformation in the MD simulation data [the dominating conformations likely present in KCl (Figure 1C)], which had a mean dye–dye distance corresponding to a much lower FRET efficiency than antiparallel conformations.

In contrast to sodium, frequent internal dynamic transitions from low to higher FRET within the same time-trace were observed in potassium conditions (Supplementary Figure S6). These frequent transitions indicate that an energy barrier for low/high FRET transitions, like the one observed in sodium buffers, is not present in potassium. Additionally, we also observed a fast equilibration in time-course folding of unlabeled Tel8 in KCl buffers measured with CD (Supplementary Figure S11) as would be expected for frequent transitions between states. These findings are a priori surprising as G-quadruplexes are much more stable in potassium than in sodium (Supplementary Figure S4), expectedly leading to less dynamics and a higher energy barrier. We propose that the many conformational states available in potassium (including hybrids, antiparallel and parallel G-quadruplexes), compared to sodium, may present a smoother energy landscape for transitioning between off-path and on-path and fully folded conformations, essentially leading to the lower energy barrier for transition and faster equilibration observed in our data. Such a model could explain the observed behavior without any negative cooperativity between consecutive G-quadruplexes, which was previously proposed to be key (43,47).

**Figure 6.**
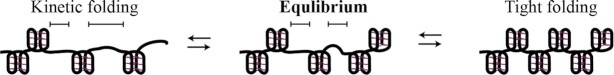
Model for long telomeric repeat G-quadruplex formation. Kinetic folding: G-quadruplexes form randomly in long telomeric DNA leaving up to three unused repeats between units noted by black lines. Tight folding: DNA folding with consecutive G-quadruplexes results in thermodynamically favored states; G-quadruplexes can fold in different conformations. Equilibrium: Due to the many partly folded states compared to the tightly packed consecutive G-quadruplexes, spacing will be present at equilibrium.

Altogether, our investigations showed that the many possible folding positions inhibit complete folding of consecutive G-quadruplexes. We presented an off-path model of consecutive G-quadruplex folding, where G-quadruplex units nucleate randomly (kinetic process) leaving up to three single-stranded repeat sequences between them and then repack to reach a more folded form. The multitude of highly stable off-path conformations lowers the energetic penalty for not forming two G-quadruplexes, resulting in an equilibrium with a dynamic population of both partly folded and fully folded conformations even in very favorable conditions for G-quadruplex folding. Our model of off-path G-quadruplex formation yields a dynamic *spaced beads-on-a-string* G-quadruplex formation (Figure 6). Increasing DNA length will likely complicate full folding even further. Spacing between G-quadruplexes (transient or long-lived) in a cellular context might leave room for loading of DNA-modifying proteins that otherwise have been suggested to be blocked by close G-quadruplex formation.

## DATA AVAILABILITY

The authors confirm that the data supporting the findings of this study are available within the article and Supplementary Data. Some parts of the raw data are available from the corresponding author, upon reasonable request.

## Supplementary Material

gkad004_Supplemental_File
