# Supplementary material for: Inhibited complete folding of consecutive human telomeric G-quadruplexes
Source: Nucleic Acids Res. 2023 Jan 30;51(4):1571–82. doi: 10.1093/nar/gkad004 (PMC9976873; doi:10.1093/nar/gkad004)
Supplement: gkad004_Supplemental_File [file gkad004_supplemental_file.pdf]

Supplementary material for:

Inhibited complete folding of consecutive human telomeric G-quadruplexes

Emil Laust Kristoffersen[1], Andrea Coletta[2], Line Mørkholt Lund[1], Birgit Schiøtt[1,2], Victoria Birkedal[1,2]*

[1] Interdisciplinary Nanoscience Center (iNANO), Aarhus University, Gustav Wieds Vej 14, 8000 Aarhus, Denmark
[2] Department of Chemistry, Aarhus University, Langelandsgade 140, 8000 Aarhus, Denmark
* To whom correspondence should be addressed: (V.B.). Tel: +4587156727; Email: vicb@inano.au.dk

CONTENTS 1
Supplementary Table S1 2
Supplementary Table S2 3
Supplementary Table S3 4
Supplementary Figure S1 5
Supplementary Table S4 6
Supplementary Table S5 6
Supplementary Figure S2 7
Supplementary Figure S3: 8
Supplementary Figure S4: 9
Supplementary Figure S5: 11
Supplementary Figure S6: 12
Supplementary Figure S7: 13
Supplementary Figure S8: 14
Supplementary Figure S10: 16
Supplementary Figure S11: 18
Supplementary Table S6 19
Supplementary Figure S12: 20
Supplementary Figure S13: 21
Supplementary Table S7 22
Supplementary Figure S14: 23
Supplementary Figure S15 25
Supplementary Figure S16 26
References: 27

Supplementary Table S1: List of DNA sequences used in this work. X is Cy5 labeled T

**Tel8**	(Bio)TTT **X**TA GGG TTA GGG TTA GGG TTA GGG TTA GGG TTA GGG TTA GGG TTA GGG TT**Cy3**
**Tel8 (unlabeled)**	TTT TTA GGG TTA GGG TTA GGG TTA GGG TTA GGG TTA GGG TTA GGG TTA GGG TT
**Tel1-4**	(Bio)TTT **X**TA GGG TTA GGG TTA GGG TTA GGG TTA TTT TTT TTT TTT TTT TTT TTT TT**Cy3**
**Tel1-4 (unlabeled)**	TTT TTA GGG TTA GGG TTA GGG TTA GGG TTA TTT TTT TTT TTT TTT TTT TTT TT
**Tel3-6**	(Bio)TTT **X**TA TTT TTT TTT TTA GGG TTA GGG TTA GGG TTA GGG TTA TTT TTT TTT TT**Cy3**
**Tel3-6 (unlabeled)**	TTT TTA TTT TTT TTT TTA GGG TTA GGG TTA GGG TTA GGG TTA TTT TTT TTT TT
**Tel5-8**	(Bio)TTT **X**TA TTT TTT TTT TTT TTT TTT TTT TTA GGG TTA GGG TTA GGG TTA GGG TT**Cy3**
**Tel5-8 (unlabeled)**	TTT TTA TTT TTT TTT TTT TTT TTT TTT TTA GGG TTA GGG TTA GGG TTA GGG TT
**Tel2-8**	(Bio)TTT **X**TA TTT TTA GGG TTA GGG TTA GGG TTA GGG TTA GGG TTA GGG TTA GGG TT**Cy3**
**Tel2-8 (unlabeled)**	TTT TTA TTT TTA GGG TTA GGG TTA GGG TTA GGG TTA GGG TTA GGG TTA GGG TT
**Tel4**	A GGG TTA GGG TTA GGG TTA GGG
**21T**	TTT TTT TTT TTT TTT TTT TTT

Supplementary Table S2: Number of experimental fluorescence time-traces with noted double-labeled constructs and salt concentration and type that showed single-step donor and/or acceptor photobleaching.

DNA construct	Salt	Conc. (mM)	Number
**Tel8**	no salt	0	48
	NaCl	25	186
		50	158
		100	165
		200	197
	KCl	25	70
		50	184
		100	135
		200	131
	LiCl	25	53
		50	55
		100	39
		200	35

DNA construct	Salt	Conc. (mM)	Number
**Tel1-4**	NaCl	100	66
	KCl	100	60
	LiCl	100	26
**Tel5-8**	NaCl	100	73
	KCl	100	79
	LiCl	100	35
**Tel3-6**	NaCl	100	101
	KCl	100	42
	LiCl	100	90
**Tel2-8**	NaCl	100	71
	KCl	100	128
	LiCl	100	29

Supplementary Table S3: Summary of the MD simulation described in the manuscript. (Hybrid-1 and Basket) were build using two consecutive (tandem) copies of the two common G-quadruplex folds: Hybrid-1 (PDB 2JSM) (1) and Basket (PDB 143D) (2). A third system (Chair) was built including two tandem copies of a "Chair" fold using as a template the mutant structure obtained by Lim *et al.* (PDB 2KM3) (3). Finally, a fourth model (Basket/Chair) was built combining the Basket and the Chair fold consecutively on the 5'- and 3'-end respectively. Note that the CTA linked for the PDB 2KM3 structure was changed back to the natural telomeric TTA sequence prior to simulations. All models were "manually" constructed linking two copies of the respective PDB structure with a -TTA- strand. To do so a 5'-GTTAG-3' single-stranded DNA (called here "linker" for short) was created with tleap (4). The "linker" structure was relaxed with a short 10 ns implicit solvent simulation. The relaxed "linker" structure was attached to the 3'-end of each of the first G-quadruplex -and a second G-quadruplex copy was attached to the 3'-end of the linker to obtain the four model tandem copies: 2JSM-TTA-2JSM, 143D-TTA-143D, mod.2KM3-TTA-mod.2K3M and 143D-TTA-mod.2K3M (named hybrid-hybrid, basket-basket, chair-chair, and basket-chair respectively in the manuscript).

SYSTEM	FOLD (PDB)	CG-MD	AA-MD
**HYBRID-HYBRID**	Hybrid-1 (2JSM)	20 μs x 2 replicas	500 ns x 2 replicas
**BASKET-BASKET**	Basket (143D)	20 μs x 2 replicas	500 ns x 2 replicas
**CHAIR-CHAIR**	Chair (mod. 2KM3)	20 μs x 2 replicas	500 ns x 2 replicas
**BASKET-CHAIR**	(143D + 2KM3)	20 μs x 2 replicas	500 ns x 2 replicas

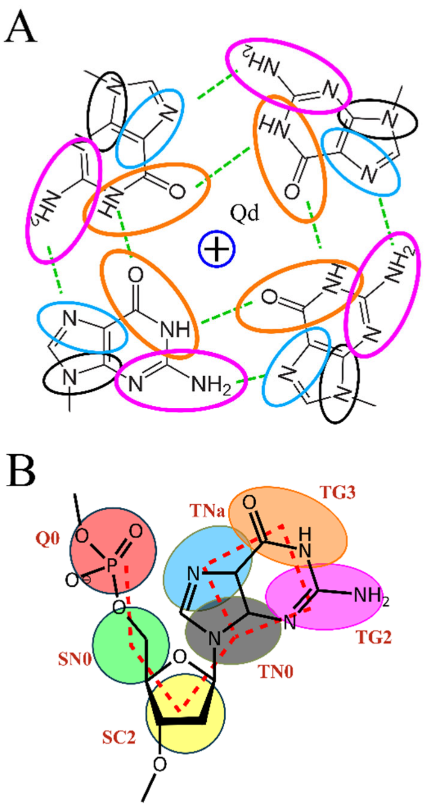

Supplementary Figure S1: A) Schematic representation of a G-quartet. The CG beads forming the Guanine side chain in the MARTINIff are indicated with Black, Purple, Cyan, and Orange ovals for TN0, TG2, TNa and TG3 beads respectively. Cations (CG bead Qd) are represented as a circle with a '+' symbol. Hoogsteen base-pairing hydrogen bonds are represented as green dashed lines. B) Schematic representation of the MARTINIff CG model of the Guanine residue. The different CG beads are represented as a colored circle (backbone) or ovals (side-chain). The connectivity between the CG beads is represented with red dashed lines.

Supplementary Table S4: Comparison between the original and the modified non-bonding parameters of some of the MARTINI coarse-grained beads involved in the formation of G-quartets (cfr. Figure S1 for the beads type). For each Bead pair, the ”type” of modification is also indicated in terms of the issue that the modification is trying to address. Ion: permit the insertion of CG ion inside the core of the G-quadruplex by reduction of the minimum distance of contact. Staking: favor the stacking between DG residues, Hoogsteen: accounts for the formation of stable hydrogen bonds in the Hoogsteen pairing.
Modifications of the force field involved the rescaling of the non-bonding interaction of monovalent ions with the TG3 beads of Guanine, as well as the stabilization of the Hogsteen base-pairing interaction by the rescaling of the non-bonding interaction between TG3, TG2, and TNa beads of Guanine. Moreover, the dihedral constraint of the original force field was substituted with a torsional potential (see Supplementary Table S5) in order to enable Guanines to explore both syn- and anti-conformation. The G-quadruplex coarse grain model included also an elastic network between the beads of the guanine side-chain in order to maintain the structure of the G-tetrad core stable.

		**Original**		**Modified**		**Type**
**Bead 1**	**Bead 2**	**σ [nm]**	**ε ($kJmol^{-1}$)**	**σ [nm]**	**ε ($kJmol^{-1}$)**	
Qd	Qd	0.47	5.0	0.35	5.0	Ion
Qd	TG2	0.47	4.0	0.32	4.0	Ion
Qd	TG3	0.47	5.0	0.32	5.6	Ion
TN0	TN0	0.32	3.5	0.32	4.0	Stacking
TN0	TNa	0.32	3.5	0.20	5.0	Stacking
TG2	TG3	0.32	2.7	0.32	5.6	Hoogsteen
TG2	TNa	0.32	4.5	0.32	5.6	Hoogsteen
TG3	TG3	0.32	2.7	0.30	5.0	Hoogsteen
TNa	TNa	0.32	4.0	0.30	4.0	Stacking

Supplementary Table S5: Parameters used in the modified MARTINI force field for the dihedral torsional potential in the form:

$$V(\varphi) = K_{\varphi} * [1 + cos(n\varphi - \varphi_s)]$$

(cfr. Figure S1 for beads type)

**Dihedral**	**φ_s (deg.)**	**K_{φ} ($kJmol^{-1}$)**	**n (multiplicity)**
Q0-SN0-SC2-TN0	0	10	2
SN0-SC2-TN0-TG2	-40	15	2
SN0-SC2-TN0-TNa	0	20	2

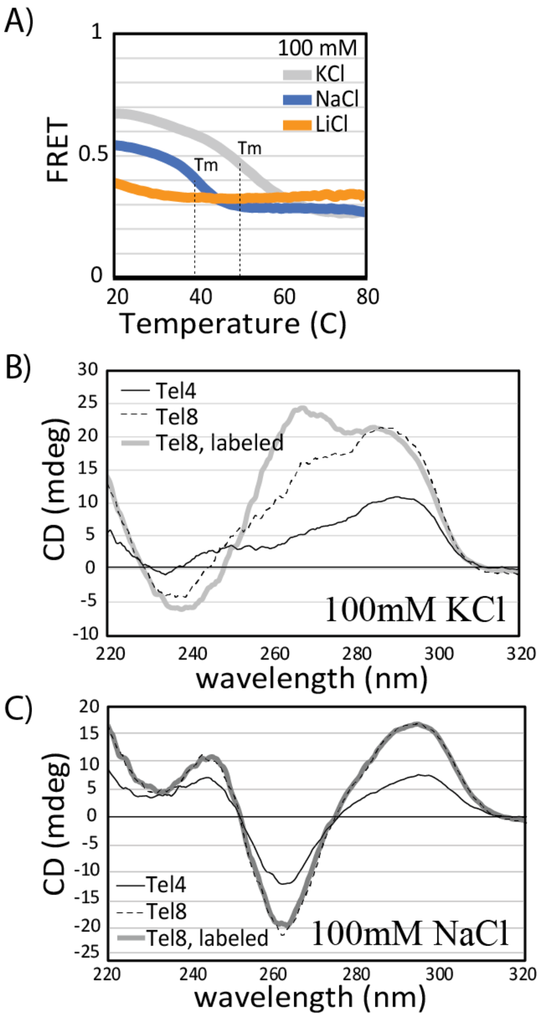

Supplementary Figure S2: A) FRET melting experiments in various salts (100 mM). Based on this, Tel8 only show melting indicating the presence of a stable structure in the presence of KCl or NaCl and not LiCl. B) and C) CD measurement of labeled and unlabeled Tel8 and unlabeled Tel4 in the presence of 100 mM KCl (B) and 100 mM NaCl (C). The difference in CD profile observed for Tel8 in KCl indicated that the presence of the fluorophore slightly changes the folding of the system in these conditions, possibly promoting formation of G-quadruplexes in the parallel conformation.

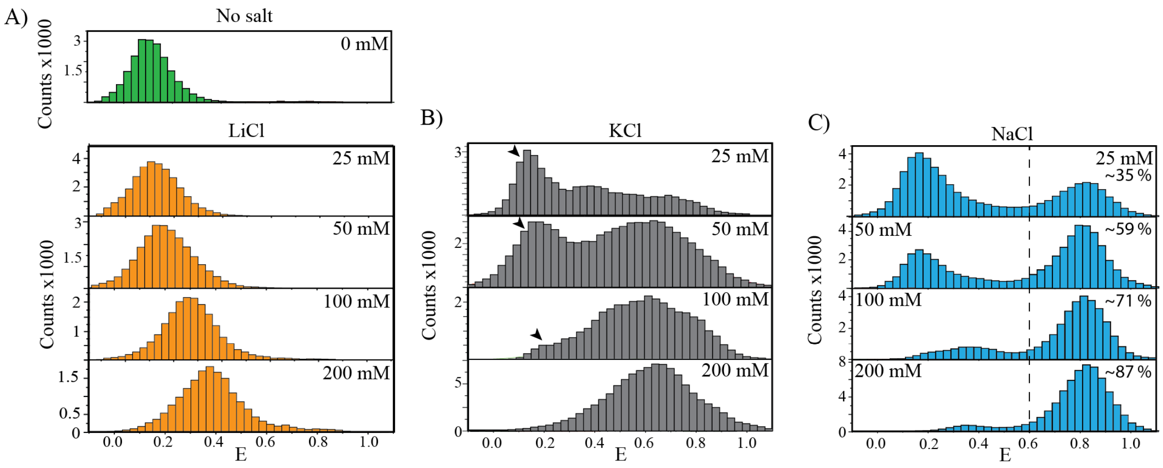

Supplementary Figure S3: Single molecule FRET histograms of Tel8 in various salts and salt concentrations in the range 0-200 mM. A) In the presence of LiCl or no salt, where G-quadruplex folding is not favored. FRET efficiencies increased with salt concentration, likely due to shielding of the backbone phosphates by Li^+ ions resulting in contraction of the worm-like unfolded DNA strand. B) In the presence of KCl that favors G-quadruplex folding, the histograms show several peaks and multiple overlapping populations of folded and unfolded conformations. A clear low FRET efficiency peak, identified as the unfolded and/or partially folded population, was observed at 25-100 mM KCl (marked by arrows). C) In the presence of NaCl, histograms show two main FRET peaks. The dashed line at E=0.6 shows our separation between the low FRET populations (unfolded or partly folded) and the high FRET populations (fully folded). The relative percentage of fully folded DNA is noted for each histogram on the figure: 35 ± 6, 59 ± 2, 71 ± 2, 87 ± 1 for 25, 50, 100 and 200 mM NaCl, respectively.

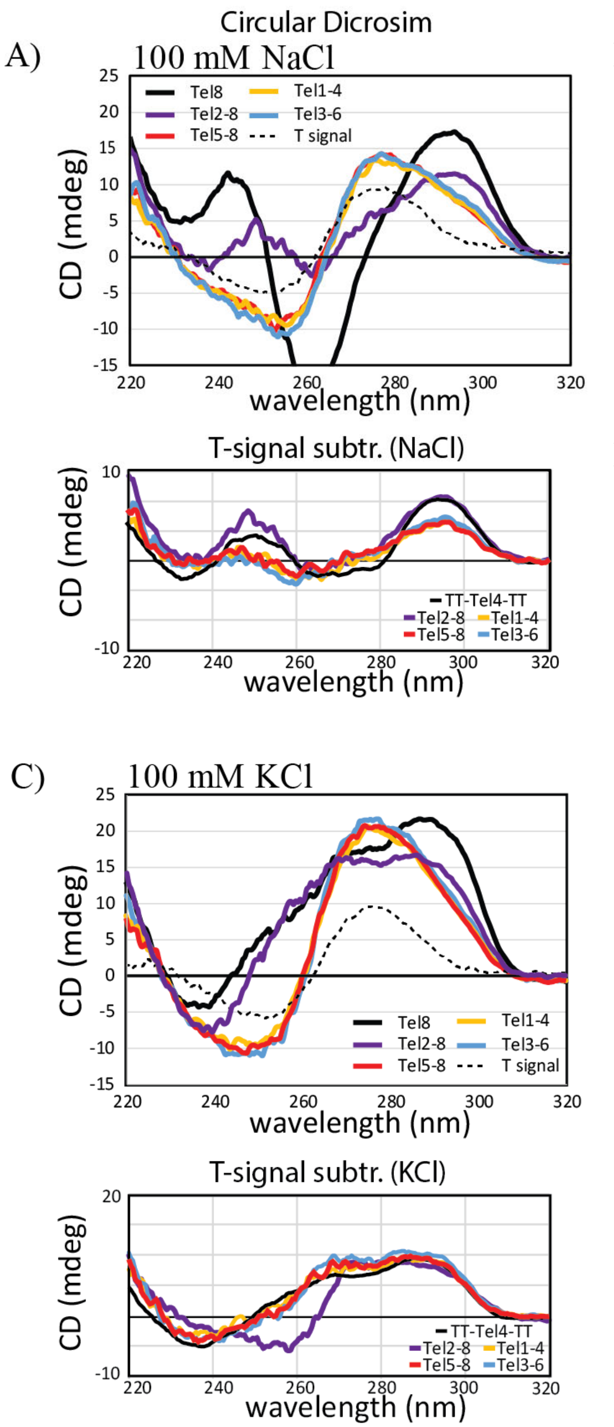

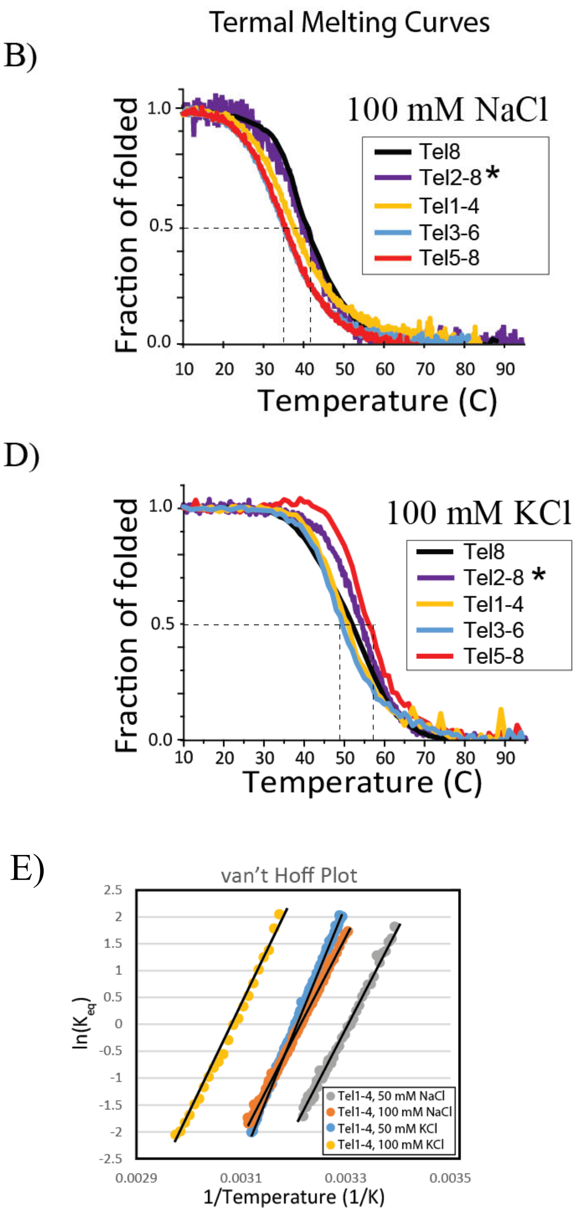

Tel1-4	ΔH (kJ/mol)	ΔS (kJ/mol)	**ΔG (kJ/mol)**
50 mM NaCl	-159	-0.53	**-4.5**
100 mM NaCl	-157	-0.51	**-8.7**
50 mM KCl	-191	-0.61	**-11.0**
100 mM KCl	-171	-0.53	**-15.9**

Supplementary Figure S4: CD spectra (A and C) and UV melting (B and D) of mutant G-quadruplex forming constructs in the presence of 100 mM NaCl (A and B) and 100 mM KCl (C and D). The CD spectra of Tel8 show typical profiles of G-quadruplex formation (A and C). The presence of additional T residues in the other constructs (especially for Tel1-4, Tel5-8, and Tel3-6) affected the recorded CD signal. The CD signal of a purely T oligonucleotide (21 T residues) is shown by the dashed black line as this component contributes to the spectra of Tel1-4, Tel5-8 and Tel3-6. In A) and C) lower

charts show plots where the T-signal has been subtracted from mutant constructs. Here the DNA construct TT-Tel4-TT (similar in sequence to Tel4 but with two additional T-residues at each end) is shown as a control for the expected CD profile. All G-quadruplex mutant constructs showed additional contributions consistent with G-quadruplex formation. Additionally, the thermal stability of these constructs is similar to that of Tel8 (see data in B and D), further supporting G-quadruplex formation. Asterisk (*) marks melting curve data that was measured using the CD spectrophotometer and not the UV spectrophotometer. E) van't Hoff plot of thermal melting curves with Tel1-4. The plots were used to calculate thermodynamic parameters in the table to the right. ΔG was calculated from the van't Hoff plot at 21 degrees Celsius (5).

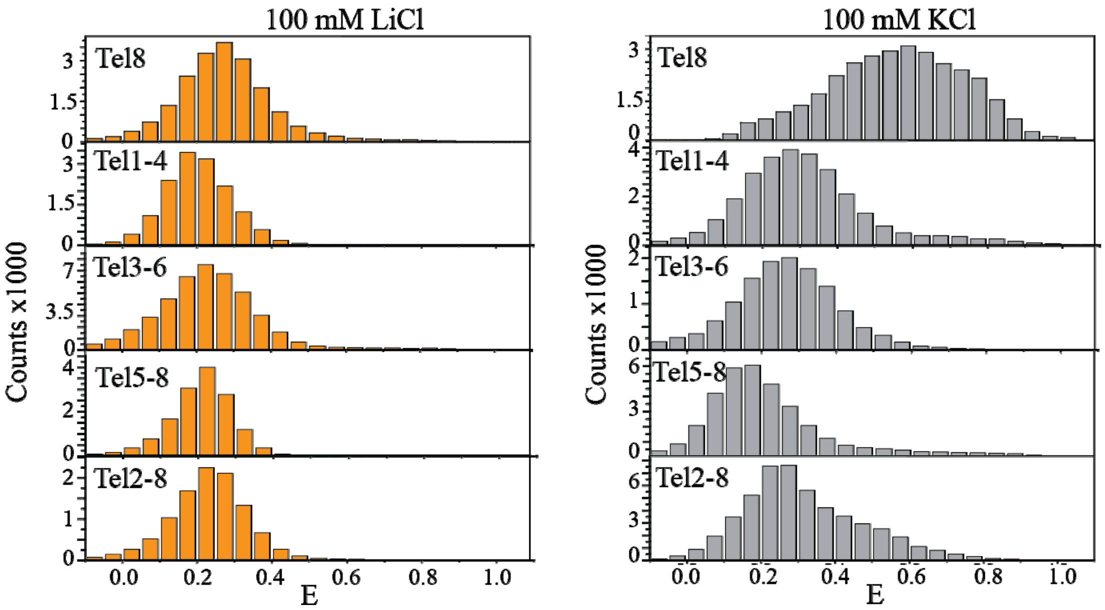

Supplementary Figure S5: Single molecule FRET histograms of mutant constructs in 100 mM LiCl (left panel) and 100 mM KCl (right panel).

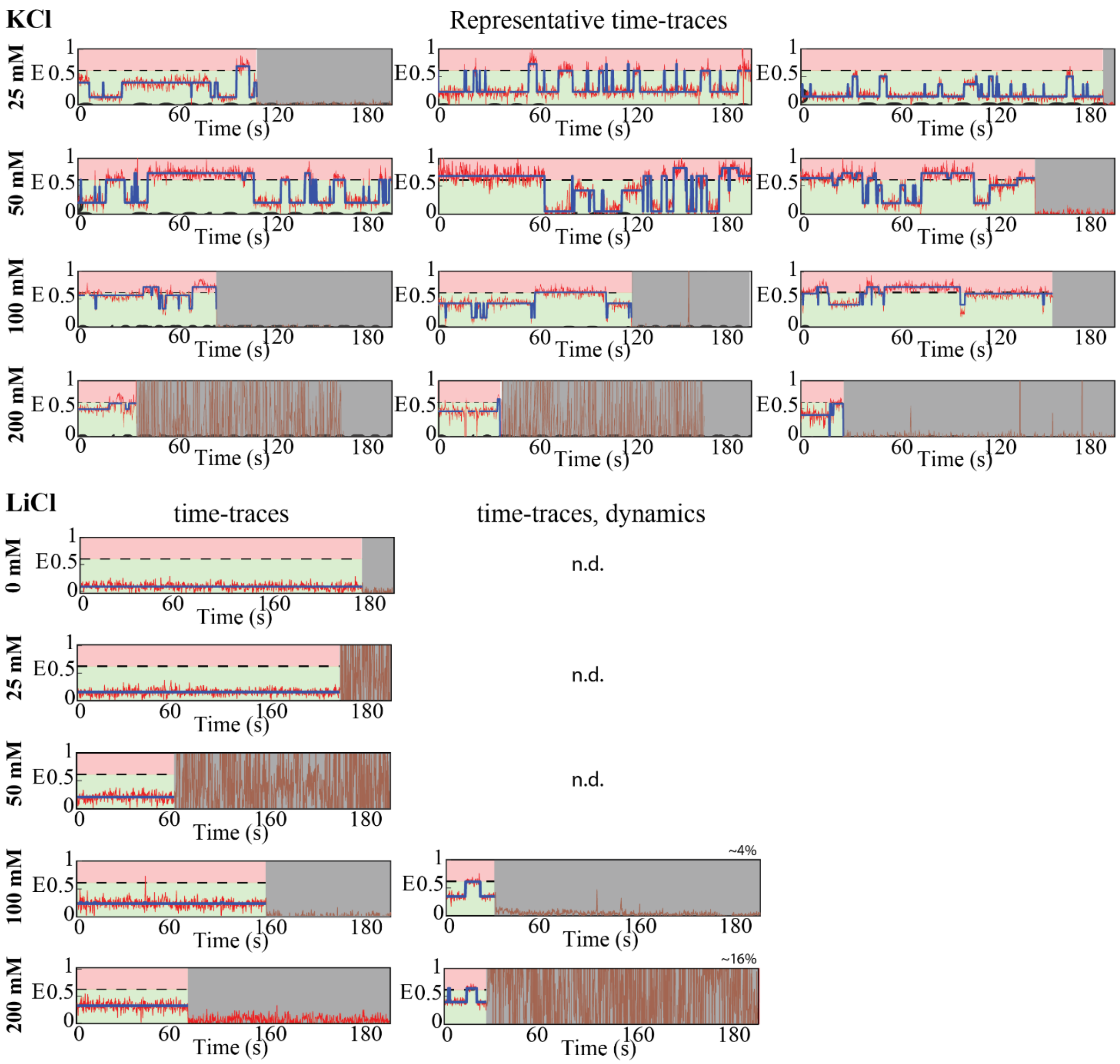

Supplementary Figure S6: Top panel: Representative single molecule FRET time-traces of Tel8 at noted KCl concentrations (in red, 3 time-traces are shown for each condition) and corresponding HMM fit (in blue). Bottom panel: Representative single molecule FRET time-traces of Tel8 at noted LiCl concentrations and corresponding HHM fit (in blue). The majority of FRET time-traces are static (left panel), few FRET time-traces show rare dynamical transitions (right panel).

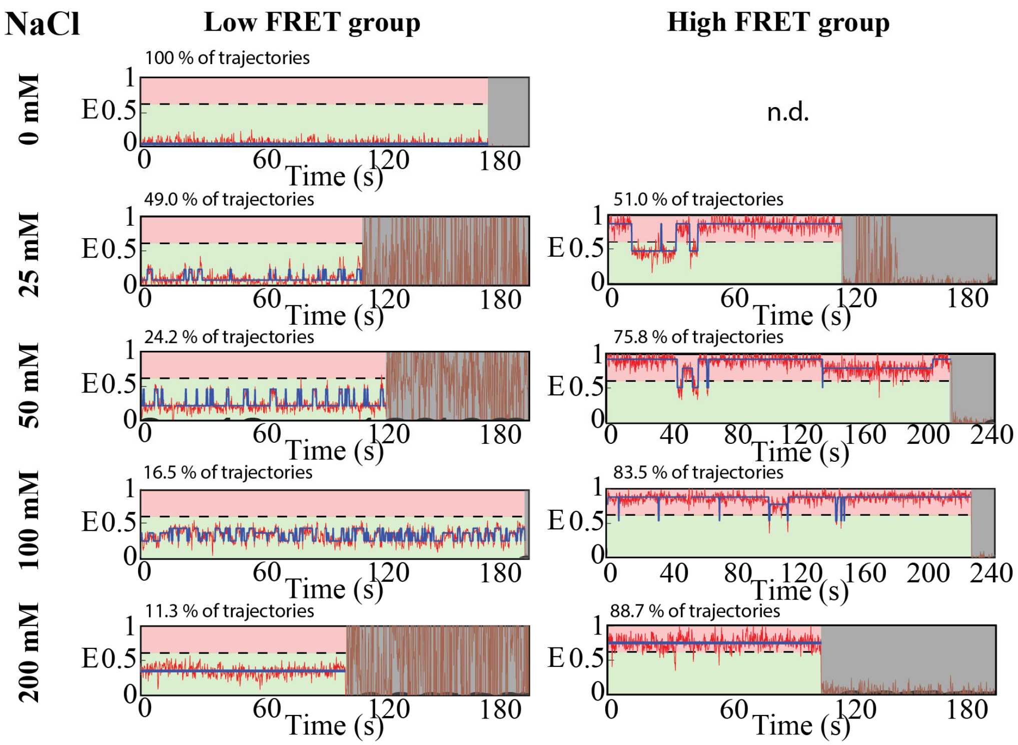

Supplementary Figure S7: Representative single molecule FRET time-traces of Tel8 at noted NaCl concentrations (in red) and corresponding HMM fit (in blue) within the low and high FRET groups defined in Section 3.3 in the main text. The percentage of FRET time-traces in each group is indicated above each representative time-trace.

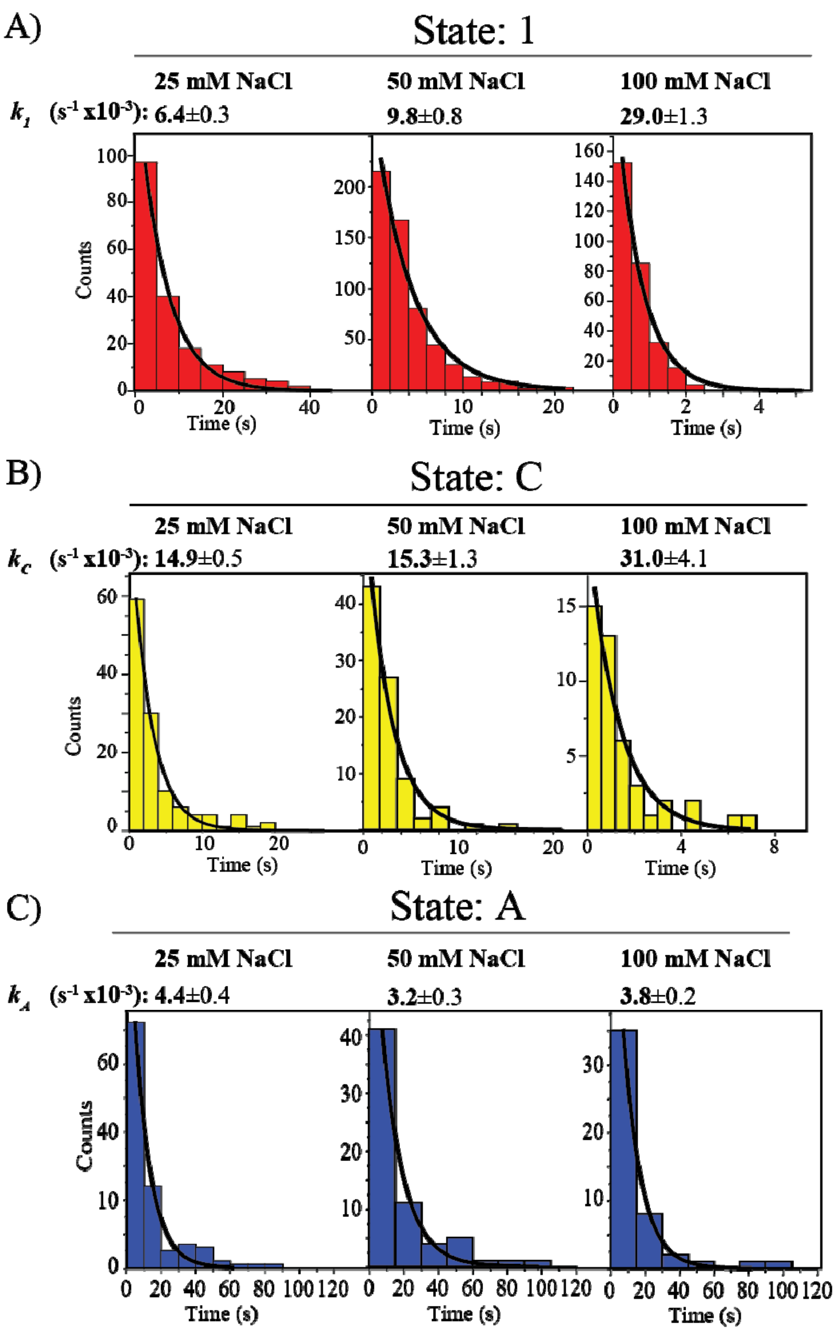

Supplementary Figure S8: A)-C) Dwell time histograms for state 1, state A and state C used for the determination of experimental rate constants, k_1, k_A and k_C, respectively. The corresponding lifetime is equal to one divided by the rate constant. Fits are shown as black lines and corresponding rate constant values are given.

A)

	NaCl		
	25 mM	50 mM	100 mM
n	6	6	4
t (s)	11437.4	11601.8	12938.4
n/t (s^{-1}x10^{-3})	**0.52**	**0.52**	**0.31**
used as k_t (s^{-1}x10^{-3})	/	**0.28**	**0.08**

B)

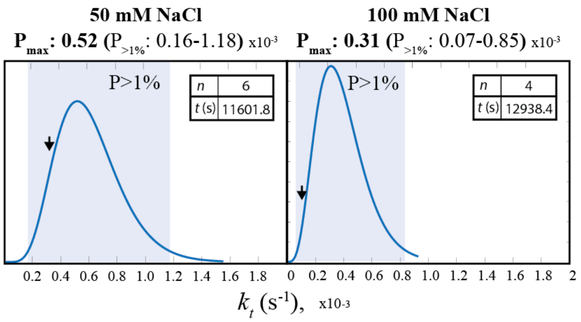

Supplementary Figure S9: A) The table quantifies the number of transitions between the lowest and high FRET states in experimental fluorescence time-traces in the high FRET group (Figure 3C in the main manuscript): number of observed transition events (*n*), total observation time in seconds (*t*) and the frequency (*n/t*), which is used to approximate the rate constant for transitions k_t. B) Plots showing the probability (*P*) of a certain rate k_t for the data in the presence of 50 and 100 mM NaCl, respectively. *P* was determined, using a Poisson distribution, as: $P_{(n\ events\ in\ timeinterval\ t)} = \frac{(k_t t)^n e^{-k_t t}}{n!}$. The peak of the curve marks the most probable k_t (P_{max}=*n/t*). The blue shading marks the range of k_ts having a probability of *P* >1% based on the observed *n/t.* The arrow marks the k_t value that was used in connection with the model at 50 and 100 mM NaCl, respectively (see Supplementary Table S6).

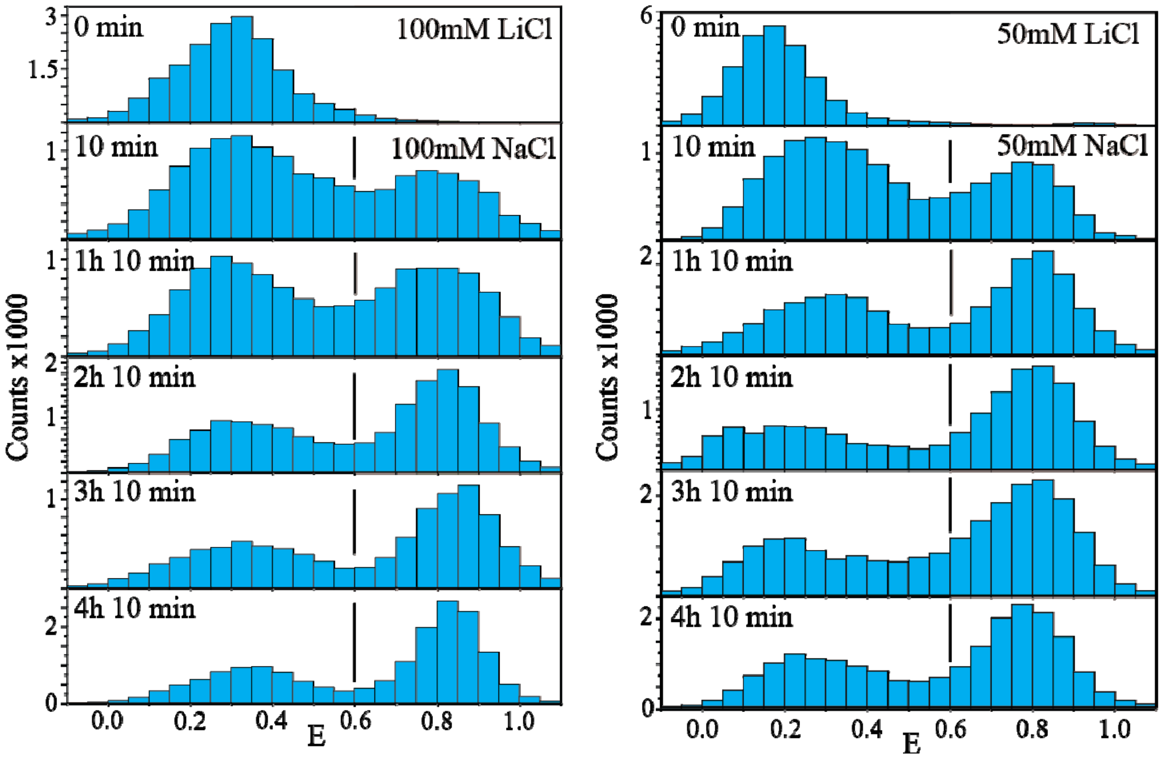

Supplementary Figure S10: Single molecule FRET histograms of time-course experiments. Time zero is taken in LiCl buffer before buffer exchange to 100 mM NaCl. E = 0.6 is marked by a black line.

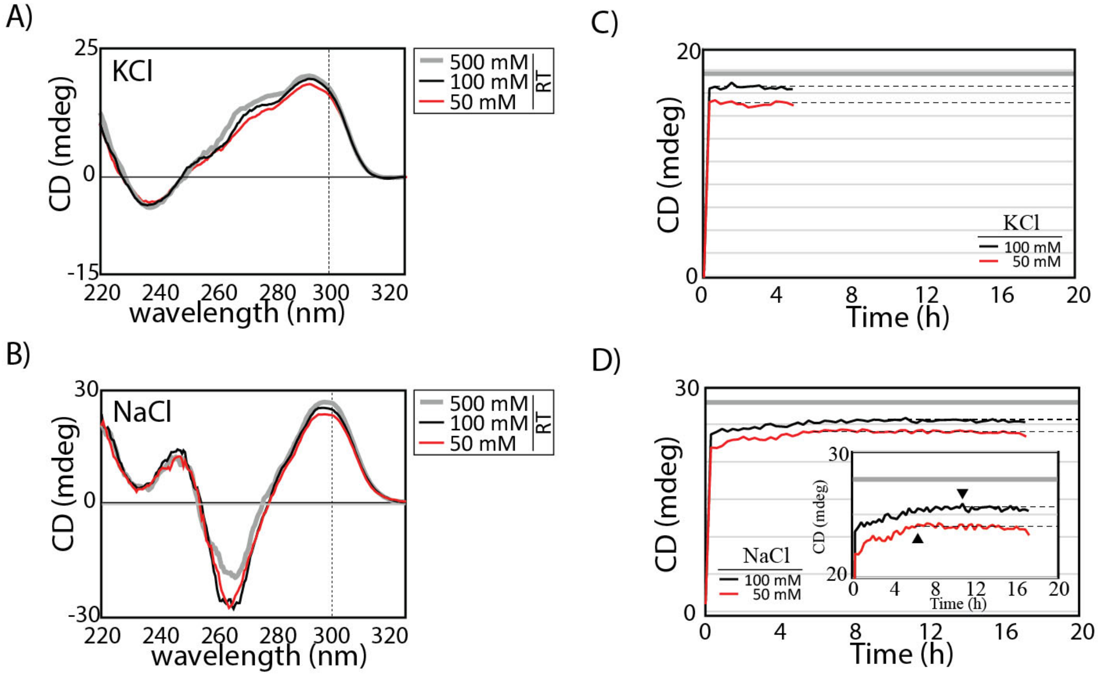

Supplementary Figure S11: Time course investigation of unlabeled Tel8 folding using CD. A) and B) Room temperature CD spectra of annealed Tel8 in noted amounts of KCl (A) and NaCl (B). C) and D) Folding kinetics of unlabeled Tel8 measured over time. The CD time course measurements were done by heating the sample to 95 °C for 5 minutes, then the temperature was set to 20 °C, and the CD signal at 295 nm was measured as a function of time until equilibrium was reached. Cuvette temperature reached room temperature after around 2.5 min. Grey lines in C and D, represent CD intensity measured at 295 nm of the annealed DNA at 500 mM KCl and NaCl, respectively.

In KCl conditions, we saw a fast folding leading to a quick equilibration. Dashed lines show the plateau values for 50 and 100 mM KCl. The final CD signal (after equilibration) was higher at 100 mM compared to 50 mM KCl.

In NaCl conditions, we saw a fast initial folding followed by slow equilibration. Also for NaCl, the final CD signal (after equilibration) was higher at 100 mM compared to 50 mM NaCl. At 50 mM NaCl, the plateau was reached after approximately 6 hours, whereas the 100 mM sample equilibrated after approximately 12 hours, see insert in panel D.

Note that the CD time-course data encompasses signal from different DNA conformations, including those with one and two folded G4s, as well as potential intermediate structures, that give a CD signal at 295 nm (6).

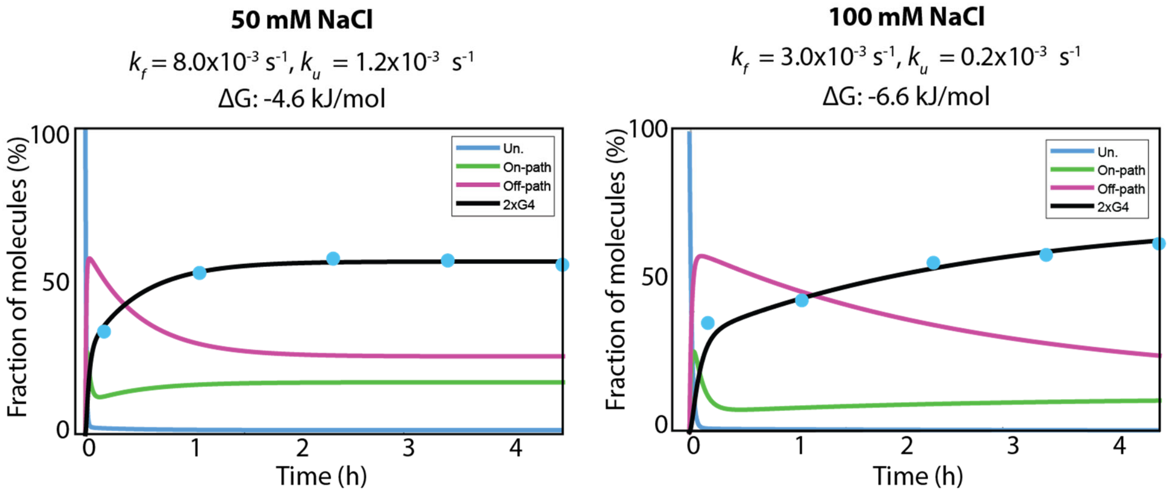

Supplementary Figure S12: Best two-parameter fit to the single molecule FRET time-course data in 50 and 100 mM NaCl, respectively (blue dots). Black line shows the fraction of the fully folded population (two G-quadruplexes (2xG4)). Green and red lines show off-path and on-path populations, respectively. The blue line shows the fully unfolded DNA population (Un.), which decreases rapidly upon folding. Fits were made using our model (see Figure 4 in the main text) setting $k_f = k_{2f}$ and $k_u = k_{2u}$. The Gibbs free energy (ΔG) was determined as $\Delta G = RT \cdot \ln(K_{eq})$, with the equilibrium constant $(K_{eq}) = k_u/k_f$. R and T are the gas constant and temperature (21 degrees Celsius), respectively.

Supplementary Table S6:
Rate constants k_f, k_{f2}, k_{u2}, and k_u were used in the off-path model (Figure 4 in the main text) and were determined from the experimentally derived reaction rates k_1, k_A, k_C, and k_t (Figure S8 and S9) as described below.
k_f: The rate constant k_f, describes the transition from unfolded DNA to one of the five one-G-quadruplex conformations (conformation II-VI as described in Figure 2 in the main text). Assuming that the experimentally derived k_1 describes the transition of unfolded DNA to any of the five one-G-quadruplex conformations, k_f was determined as follows: $k_f = k_1/5$.
k_u: The rate constant k_u describes the unfolding of any of the five one-G-quadruplex conformations to unfolded DNA. We used k_t values (Figure S9), assuming that k_t represents the reaction rate of going from an off-path conformation - via the unfolded state - to the fully folded conformation. Since folding is faster than unfolding when salt is present, the rate constant for unfolding is assumed rate-limiting and thus k_t is dominated by k_u. However, unfolded DNA only has a 2/5 chance of forming on-path conformations leading to full folding giving $k_t \approx k_u \cdot (2/5)$. Given that this is an approximation and that there is a significant experimental uncertainty linked to the determination of k_t, we tried out different values of k_u and selected those that described the data well (see Figure 4A in the main text and arrows in Figure S9).
k_{f2}: The rate constant k_{f2} describes the folding from the on-path G-quadruplexes (conformations II or VI as described in Figure 2 in the main text) to the fully folded conformation and was determined as: $k_{f2} = k_C$.
k_{u2}: The rate constant k_{u2} describes the unfolding of the fully folded conformation into one of the two on-path one-G-quadruplex conformations (conformations II or VI). Assuming that k_A gives the rate of unfolding into any of the two on-path conformations, k_{u2} was determined as $k_{u2} = k_A/2$. Contributions from state B were neglected as state A is dominant (see section 3.3 in the main text).

		Rate constant (s^{-1} x10^{-3})	
	NaCl	50 mM	100 mM
1st G4*	k_f	**2.0**±0.2	**5.8**±0.3
	k_u	**0.7**	**0.2**
2nd G4*	k_{f2}	**15.3**±1.4	**31.0**±4.1
	k_{u2}	**1.6**±0.2	**1.9**±0.1

* G4: G-quadruplex

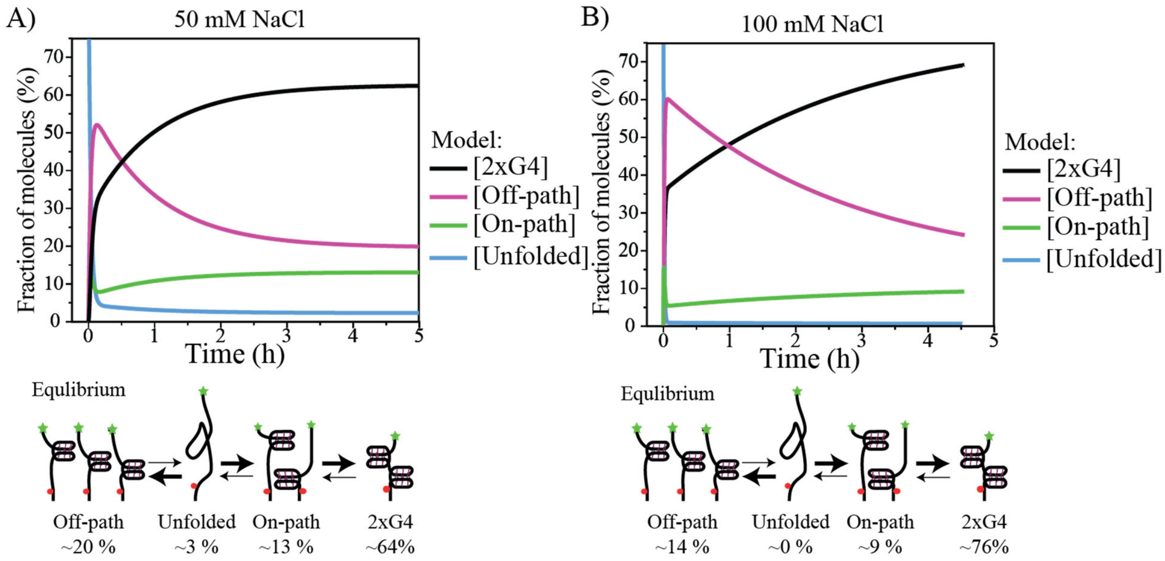

Supplementary Figure S13: A) and B) Fractions of fully folded (2xG4, black), off-path, (purple), on-path (green), and unfolded (blue) structures given by our off-path model (see Figure 4 in the main text) as a function of time using parameters from Table S6 in 50 mM and 100 mM NaCl, respectively. Cartoon illustrations below charts show the percent proportion of the different subpopulations at equilibrium.

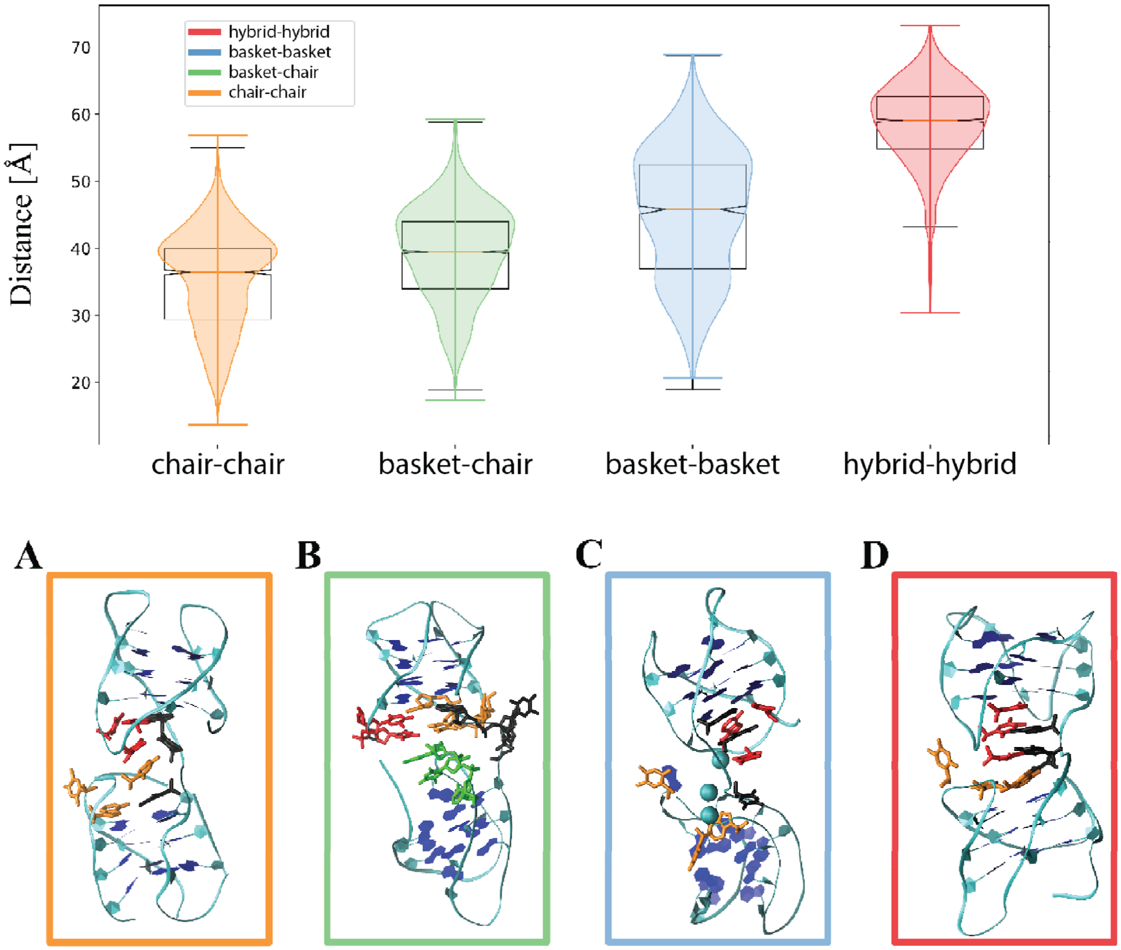

Supplementary Figure S14: Distributions of the average distance of dye accessible volumes calculated over conformations sampled by Coarse-Grained G-quadruplex simulations of the four G-quadruplex-tandem systems. Top panel, Violin plot overlaid on box plot of accessible volume distances (indicated in Å on the left-side y-axis) for the various system: in yellow the chair-chair system, in green the mixed basket-chair, in blue the basket-basket and in red the hybrid-hybrid; on the right-side y-axis shows the FRET efficiency when assuming R_0=52 Å. Bottom Panel, centroid structure of the largest structural clusters observed in the AA-MD simulations of the four systems (the box color outline follows the same color scheme described for the top panel). DNA is represented as cartoon, connecting loops at the interface between the two G-quadruplexes are represented as licorice (A: L0 in orange, L4 in black, L6 in red B: L2 in green, L4 in black, L6 in orange L8 in red C: L2 in orange, L4 in black, L6 in red, D: L2 in orange, L4 in black, L7 in red)

Supplementary Table S7: Average distance of dye accessible volumes calculated on the two largest clusters extracted from CG-MD simulations (indicated as CG-MD CL.1 and 2), the parameter of the two Gaussian-fit of the accessible volume distributions reported in Figure 5 in the manuscript (indicated as G1 and G2) and the average distance calculated for the two AA-MD replica simulations. For CG-MD clusters, the percentage of total analyzed frames in the respective clusters are reported in parentheses. *: in these cases, CL.1 is the union of the two largest clusters and CL.2 is the third largest cluster.
The relative dye-dye distances obtained from full AA-MD simulations were very similar, but slightly shorter compared to those observed in CG-MD. This may be explained by a stronger non-bonding potential of the AA-force field when compared with the CG-force field leading to more compact structures.

SYSTEM	CG-MD CL.1 DIST [Å]	CG-MD CL.2 DIST [Å]	G1	G2	AA-MD0 DIST[Å]	AA-MD1 DIST[Å]
**hybrid-hybrid**	59 ± 7 (41%)*	60 ± 5 (7%)	57 ± 6 (63%)	62 ± 4 (35%)	44±6	55 ± 6
**basket-basket**	49 ± 7 (20%)	35 ± 7 (17%)	50 ± 6 (68%)	34 ± 4 (33%)	37±7	45 ± 11
**chair-chair**	37 ± 6 (52%)*	27 ± 7 (8%)	39 ± 4 (55%)	28 ± 6 (43%)	33±6	22 ± 9
**basket-chair**	42 ± 5 (17%)	35 ± 5 (15%)	37 ± 7 (71%)	43 ± 4 (29%)	32 ± 9	23 ± 9

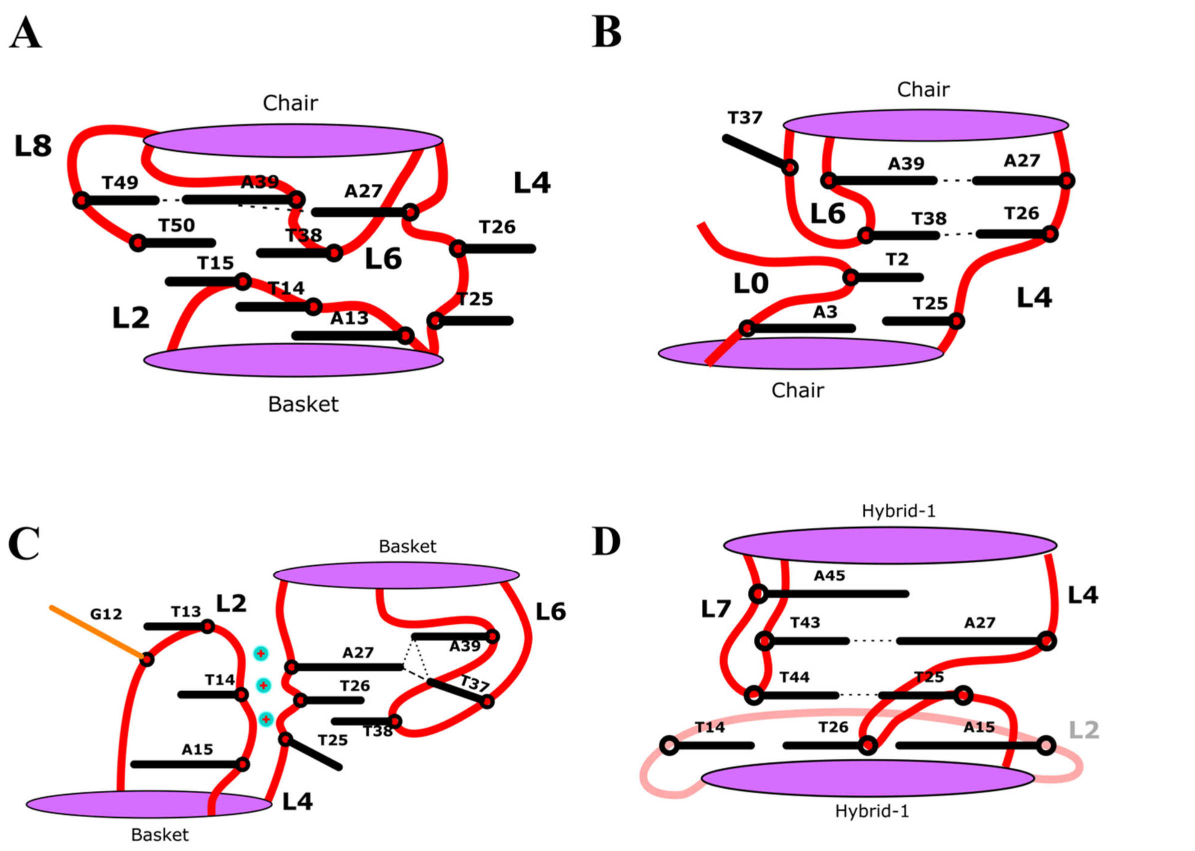

Supplementary Figure S15: Summary of results from G-quadruplexes *in-silico* modeling. Schematic representation of the interface between the tandem G-quadruplexes observed in the centroid structure of the largest cluster observed in the all-atom MD simulations. A) Mixed chair-basket system B) chair-chair system C) basket-basket system D) hybrid-hybrid system.

In all schemes the G-quartet facing toward the center of the tandem are reported as purple discs; the phosphate backbone or the –TTA- loops are represented; the nucleobase side-chain are represented as straight lines (black for Adenine or Thymine, Orange for Guanine). The Nucleobase type (A for Adenine T for Thymine and G for Guanine) and number in the sequence are also indicated, together with the Loop number (see Figure 1 in the manuscript). Cations observed in close contact with the DNA backbone are reported as cyan full circles with a “+” sign.

In the hybrid-hybrid system, the nucleobases A27:T43 (from L4 and L7 respectively) form a reversed Hoogsteen pairing while T25:T44 form a symmetric homo-pyrimidine pairing (see panel D).

The basket-basket system shows a less stable relative orientation of the two G-quadruplexes, compared to hybrid-hybrid, and the two G-quadruplex folds appear more “disconnected”. Only a few stable hydrogen bonds and stacking interactions are observed between L4 and L6. This is likely a result of the baskets system having two crossing loops (L2 and L6) facing the interphase, which hinders the formation of stable interactions. We observed that only L6 forms nucleobase mediated interaction with L4 while L2 forms a backbone mediated interaction requiring Na^+ to screen the phosphate charges (A27:T37 form a reversed Watson-Crick paring while A39 forms hydrogen bonds with both A27 and T37 causing a slight deformation of the reversed Watson-Crick paring in A27:T37, see Figure S11C). A further indication of the instability of this system is given by the fact that in the first replica simulation, the guanine base G12 had detached from the G-quartet (highlighted in Orange in panel C).

The chair-chair system shows a compact interaction between L4, L6, and the 5’ single-strand overhang (L0) (A27:A39 form a symmetric homo-purine pairing, T26:T38 form a symmetric homo-pyrimidine pairing similar to that observed for T25:T44 in Hybrid-1, see panel B). Like for hybrid-hybrid conformations, the chairs system had a narrower dye-dye size distribution (Supplementary Figure S13), at least for the main cluster population, which might be due to the stabilizing interactions.
The basket-chair system (see panel A) also shows a compact interaction involving stacking and paring of nucleobases from L2, L4, L6, and L8. Here a planar triplet formed by a Watson-Crick pairing between A39:T49 and an asymmetric homo-purine between A39:A27 is stacking right below the G-quartet at the interface between the two G-quadruplex. Note that in all systems A27, part of the connecting loop L4 seemed to play a central role.

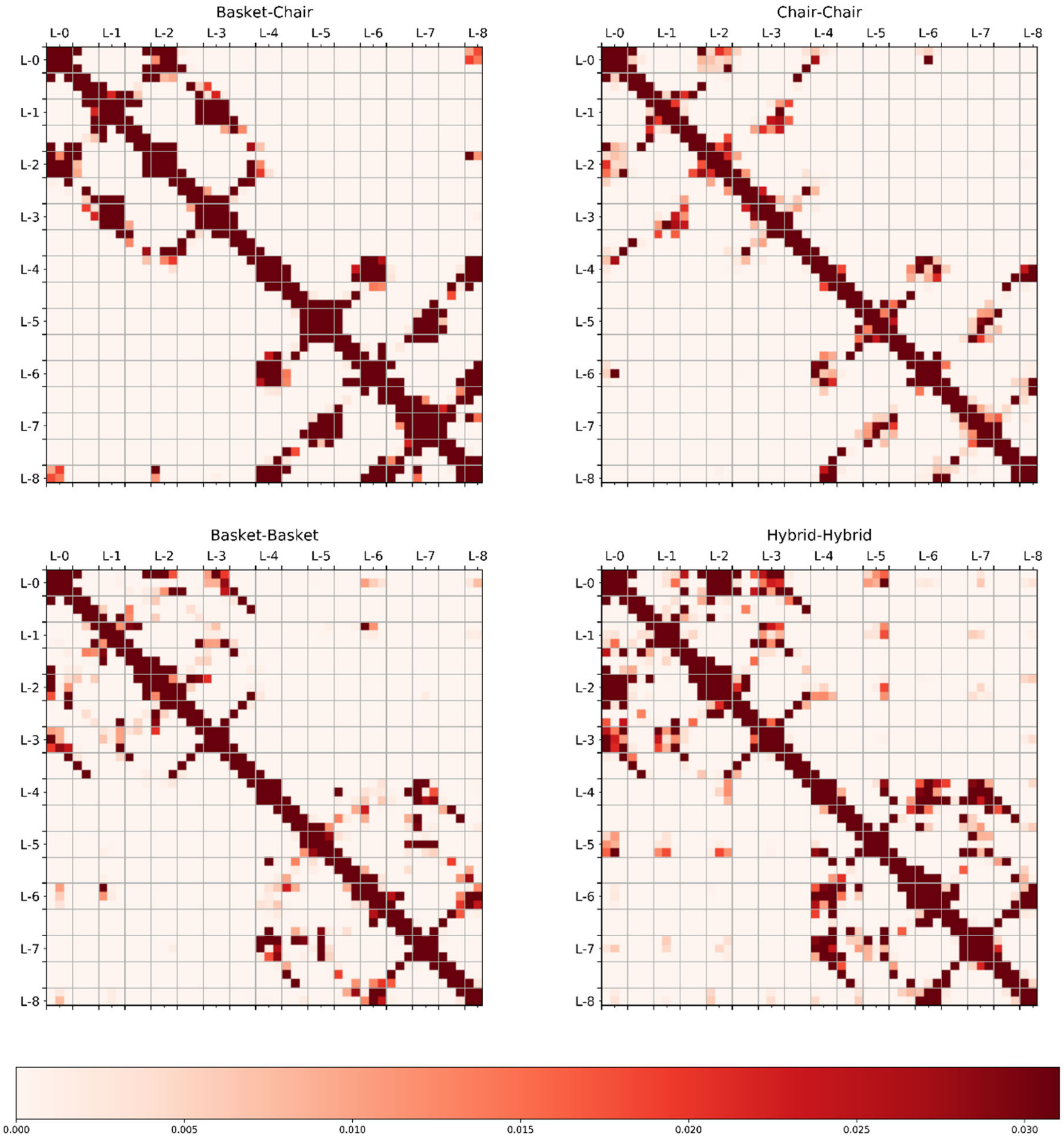

Supplementary Figure S16: Per-residue probability of contact between -TTA- loop nucleobases in the CG-MD simulation of the four tandem G-quadruplex systems. The loop number is indicated accordingly to Figure 1 in the main text. Residues were considered in contact if the minimum distance between any residue beads was shorter than 3.5 Angstrom.

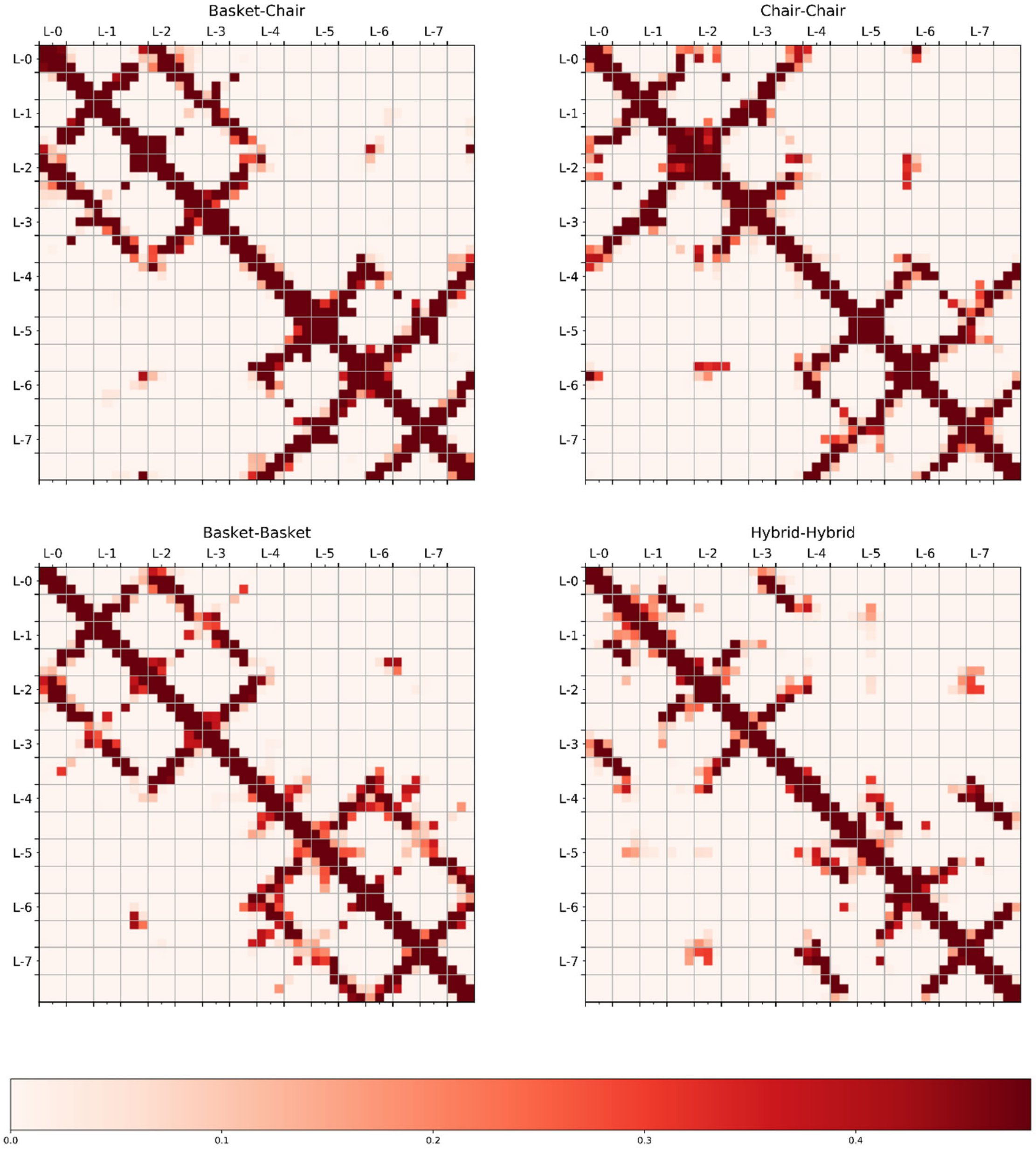

Supplementary Figure S17: Per-residue probability of contact between -TTA- loop nucleobases in the AA-MD simulation of the four tandem G-quadruplex systems. The loop number is indicated accordingly to Figure 1 in the main text. Residues were considered in contact if the minimum distance between any residue beads was shorter than 3.5 Angstrom.

References:

1. Phan,A.T., Kuryavyi,V., Luu,K.N. and Patel,D.J. (2007) Structure of two intramolecular G-quadruplexes formed by natural human telomere sequences in K+solution. *Nucleic Acids Res.*, **35**, 6517–6525.
2. Wang,Y. and Patel,D.J. (1993) Solution structure of the human telomeric repeat d[$AG_3(T2AG_3)_3$] G-tetraplex. *Structure*, **1**, 263–282.
3. Lim,K.W., Alberti,P., Guédin,A., Lacroix,L., Riou,J.F., Royle,N.J., Mergny,J.L. and Phan,A.T. (2009) Sequence variant (CTAGGG)n in the human telomere favors a G-quadruplex structure containing a G·C·G·C tetrad. *Nucleic Acids Res.*, **37**, 6239–6248.
4. D.A. Case, J.T. Berryman, R.M. Betz, D.S. Cerutti, T.E. Cheatham, III, T.A. Darden, R.E. Duke,T.J.G., H. Gohlke, A.W. Goetz, N. Homeyer, S. Izadi, P. Janowski, J. Kaus, A. Kovalenko, T.S. Lee,S.L., P. Li, T. Luchko, R. Luo, B. Madej, K.M. Merz, G. Monard, P. Needham, H. Nguyen, H.T. Nguyen,I., Omelyan, A. Onufriev, D.R. Roe, A. Roitberg, R. Salomon-Ferrer, C.L. Simmerling, W. Smith,J.S. and R.C. Walker, J. Wang, R.M. Wolf, X. Wu,D.M.Y. and P.A.K. (2015) AmberTools15, University of California, San Francisco.
5. Mergny,J.L. and Lacroix,L. (2009) UV melting of G-quadruplexes. *Curr. Protoc. Nucleic Acid Chem.*, **chap. 17**, 17.1.1-17.1.15.
6. Koirala,D., Mashimo,T., Sannohe,Y., Yu,Z., Mao,H. and Sugiyama,H. (2012) Intramolecular folding in three tandem guanine repeats of human telomeric DNA. *Chem. Commun.*, **48**, 2006–2008.
